# Young and undamaged recombinant albumin alleviates T2DM by improving hepatic glycolysis through EGFR and protecting islet β cells in mice

**DOI:** 10.1186/s12967-023-03957-3

**Published:** 2023-02-06

**Authors:** Hongyi Liu, Anji Ju, Xuan Dong, Zongrui Luo, Jiaze Tang, Boyuan Ma, Yan Fu, Yongzhang Luo

**Affiliations:** 1 School of Life Sciences, Tsinghua University, Beijing, 100084 China; 2 Tsinghua-Peking Joint Center for Life Sciences, Beijing, 100084 China; 3 The National Engineering Research Center for Protein Technology, Beijing, 100084 China; 4 Beijing Key Laboratory for Protein Therapeutics, Beijing, 100084 China; 5 Present Address: School of Life Sciences, Tsinghua University, Beijing, 100084 China

**Keywords:** rMSA, Type 2 diabetes mellitus, Glycolysis, β-Cell apoptosis, Lipotoxicity

## Abstract

**Background:**

Albumin is the most abundant protein in serum and serves as a transporter of free fatty acids (FFA) in blood vessels. In type 2 diabetes mellitus (T2DM) patients, the reduced serum albumin level is a risk factor for T2DM development and progression, although this conclusion is controversial. Moreover, there is no study on the effects and mechanisms of albumin administration to relieve T2DM. We examined whether the administration of young and undamaged recombinant albumin can alleviate T2DM in mice.

**Methods:**

The serum albumin levels and metabolic phenotypes including fasting blood glucose, glucose tolerance tests, and glucose-stimulated insulin secretion were studied in *db*/*db* mice or diet-induced obesity mice treated with saline or young, undamaged, and ultrapure rMSA. Apoptosis assays were performed at tissue and cell levels to determine the function of rMSA on islet β cell protection. Metabolic flux and glucose uptake assays were employed to investigate metabolic changes in saline-treated or rMSA-treated mouse hepatocytes and compared their sensitivity to insulin treatments.

**Results:**

In this study, treatment of T2DM mice with young, undamaged, and ultrapure recombinant mouse serum albumin (rMSA) increased their serum albumin levels, which resulted in a reversal of the disease including reduced fasting blood glucose levels, improved glucose tolerance, increased glucose-stimulated insulin secretion, and alleviated islet atrophy. At the cellular level, rMSA improved glucose uptake and glycolysis in hepatocytes. Mechanistically, rMSA reduced the binding between CAV1 and EGFR to increase EGFR activation leading to PI3K-AKT activation. Furthermore, rMSA extracellularly reduced the rate of fatty acid uptake by islet β-cells, which relieved the accumulation of intracellular ceramide, endoplasmic reticulum stress, and apoptosis. This study provided the first clear demonstration that injections of rMSA can alleviate T2DM in mice.

**Conclusion:**

Our study demonstrates that increasing serum albumin levels can promote glucose homeostasis and protect islet β cells, which alleviates T2DM.

**Supplementary Information:**

The online version contains supplementary material available at 10.1186/s12967-023-03957-3.

## Background

Type 2 diabetes mellitus (T2DM) has become a severe disease that has reached epidemic proportions and thus poses an increasing threat to public health as socioeconomic status improves [1]. As a result, it is critical to investigate the mechanisms behind the onset and progression of T2DM, as well as to develop effective medicines to reduce or possibly cure diabetes.

In T2DM, insulin resistance causes changes in glucose metabolism, resulting in hyperglycemia [2]. The liver plays a central role in systemic glucose homeostasis [3], which is controlled by insulin [4]. Insulin signals via the PI3K-AKT pathway inhibits gluconeogenesis and activates glycolysis. AKT inhibits the expression of gluconeogenesis genes by inhibiting FOXO1 [5], meanwhile, AKT promotes glycolysis by activating glucokinase [6]. Therefore, the interaction between the insulin signals and glucose flux regulates hepatic glucose metabolism. Besides the reduced insulin sensitivity, β-cell apoptosis is a crucial component of T2DM pathogenesis [7]. Free fatty acid (FFA) over-uptake that mediates lipotoxicity can cause β-cell dysfunction by causing endoplasmic reticulum (ER) stress [8]. In this process, ceramide formed from saturated FFA is an essential substance in islet β-cell ER stress and apoptosis [9].

Albumin is the most abundant protein in serum and serves as a transporter of FFA in blood vessels and intercellular stroma, enabling FFA transport across organ tissues such as the liver, fat, heart, and skeletal muscle [10]. In T2DM patients, serum albumin levels can be decreased by impaired albumin synthesis [11], glycation-caused immune response-mediated clearance [12], and albuminuria [13]. Serum albumin levels are significantly reduced in some T2DM patients [13–15] and have been shown to be a risk factor for T2DM development and progression [16–18], although this conclusion is controversial [19]. Nevertheless, there has been, to date, no report that increasing the serum albumin level can improve T2DM. Noteworthily, the uncertainty in the quality of blood-derived products can bring many risks to the treatment of T2DM; for example, the glycated HSA has negative effects on patients and limits the value of blood-derived HSA in the treatment of T2DM [13]. Moreover, commercial blood-derived HSA products have been reported to have the disadvantage of decreased functions and causing immunosuppression [20, 21]. Fortunately, our group reported that long-term administration of young, undamaged, and ultrapure recombinant MSA (rMSA) can extend the lifespan and healthspan of mice without side effects [22]. Mechanically, the rMSA is significantly superior to blood-derived MSA in the free thiol, AGE, carbonylation, and homocysteine levels, which is extremely important for its functions [22]. Here, we report that rMSA can enhance glycolysis through the EGFR-PI3K-AKT signaling in hepatocytes and protect islet β-cells from apoptosis. On this basis, we discovered for the first time that injecting rMSA improved blood glucose homeostasis in T2DM mice.

## Material and methods

### Mice and drug treatments

Wild-type SPF-grade male C57BL/6J male mice (WT) were purchased from Vital River Experimental Animal Technology Co., LTD. SPF-grade Male *B6.Cg-dock7m Lepr*^*db*^/++/*J* (*db*/*db*) mice (strain#: 000699) were purchased from The Jackson Laboratory (JAX). The mice were professionally and securely delivered to Laboratory Animal Research Center, Tsinghua University (THU-LARC). The mice were kept stable for a week before undergoing the studies to ensure that they acclimated to their new habitat. The animals were housed in a sterile barrier environment at 23 °C with a 12-h cycle of light and darkness. After arrival, mice on a normal diet (ND) were fed with irradiation-sterilized JAX-standard breeder chow (SHOOBREE®, Xietong Pharmaceutical Biotechnology Co., Ltd., 1010058) and sterilized water during the entire study. An adequate number of 8-week-old WT mice were chosen for diet-induced obesity (DIO) and fed a high-fat diet (HFD; ResearchDiet, D12492) for 8 weeks. Mice with a bodyweight of more than 30 g were chosen to participate in the experiments.

The young, undamaged, and ultrapure rMSA and treatments used in this study were the same as previously described [22] unless stated otherwise herein. Briefly, mice in the rMSA-treated group were *i.v.* injected once every 3 weeks with rMSA in a dose of 1.5 mg per gram of body weight, while the control group mice were injected with isometric saline. All animal studies were approved by the Institutional Animal Care and Use Committee of Tsinghua University (Beijing, China).

### Cell culture, treatments, and transfections

Mouse hepatocytes AML12 were purchased from ATCC (#CRL-2254) and cultured with DMEM/F12 medium (Gibco) supplemented with 10% FBS, 10 μg/ml insulin, 5.5 mg/ml transferrin, 5 ng/ml selenium, 40 ng/ml dexamethasone, and 15 mM HEPES. Mouse insulinoma cell line MIN6 (Beijing Crisprbio, CE18728) was maintained under a 5% CO_2_ atmosphere at 37 °C in RPMI 1640 medium supplemented with 10% FBS, 10 mM HEPES, 2 mM l-glutamine, 1 mM sodium pyruvate, and 0.05 mM 2­mercaptoethanol. Mouse primary hepatocyte isolation was performed as previously described [23].

For mass labelling of AML12 hepatocytes, AML12 hepatocytes were washed twice with warm PBS and starved in serum-free DMEM overnight. Labelled compounds [U-^13^C]-glucose (Sigma, 389374) were added to serum-free DMEM (without glucose). Indicated concentrations of rMSA with or without insulin were added at the same time.

For signaling pathway analysis, AML12 hepatocytes or mouse primary hepatocytes were washed twice with warm PBS and starved in serum-free DMEM overnight. For the AKT signaling, after the pretreatment with vehicle, Wortmannin (Selleck, S2758), PI-103 (Selleck, S1038), or ZSTK474 (Selleck, S1072), respectively for 30 min, indicated concentrations of rMSA with or without insulin were added at the same time in the absence or presence of PI3K inhibitors. For the IGF-1R signaling, after the pretreatment with vehicle or OSI-906 (Selleck, S1091) for 30 min, indicated concentrations of rMSA with or without insulin were added at the same time in the absence or presence of IGF-1R inhibitor. For the SRC signaling, after the pretreatment with vehicle, PP1 (Selleck, S7060), or Dasatinib (Selleck, S1021) for 30 min, indicated concentrations of rMSA were added at the same time in the absence or presence of SRC inhibitors. For the EGFR signaling, after the pretreatment with vehicle, BDTX-189 (Selleck, S9786), or Varlitinib (Selleck, S2755) for 30 min, indicated concentrations of rMSA were added in the absence or presence of EGFR inhibitors.

For induction of MIN6 cell apoptosis, sodium palmitate (PA) was dissolved to 250 mM in 50% ethanol at 60 °C, conjugated to 10% fatty acid-free bovine serum albumin (BSA; Beyotime, ST025), and added to serum-free RPMI 1640 medium to a final concentration of 0.2 mM PA and < 0.05% ethanol. PA, rMSA, sulfo-*N*-succinimidyl oleate sodium (SSO; MCE, HY-112847A), Fumonisin B1 (MCE, HY-N6719), CCT020312 (MCE, HY-119240), and GSK2656157 (Selleck, S7033) of specific concentrations were dissolved as indicated or manufacturer’s instructions and used to treat MIN6 cells for 16 h.

For immobilized rMSA and PA treatment, NHS-activated agarose (Sangon Biotech, C600024) was used to bind rMSA following the manufacturer’s instruction. Briefly, the agarose was washed and balanced with 1 mM HCl and sodium bicarbonate buffer (pH = 8.0) respectively. Then rMSA and the agarose, or the agarose alone, were co-incubated overnight in sodium bicarbonate buffer (pH = 8.0) at 4 °C, following blocking with 0.1 M Tris–HCl Buffer (pH = 8.5). FITC labeled rMSA was also immobilized with the same method to examine whether the rMSA could be dissociated from the agarose and uptake by cells. MIN6 cells were cultured in 6-well plates for 80% confluence. PA or vehicle, and immobilized rMSA or empty agarose were added to Transwell chambers with 4 μm pore membranes hanging above the cells. Cells were treated for 16 h and subjected to western blot for apoptosis assessment.

Transfection was performed with FuGene HD (Roche Applied Science). Cav1 (5′-CUGCGAUCCACUCUUUGAATT-3′ and 5′-CGCUUGUUGUCUACGAUCUTT-3′), and control small interfering RNAs (siRNAs) were from Genechem.

### Assessment of glucose homeostasis in animals

Mice fasted for 6 h before fasting blood glucose level (FBGL) values were measured using a glucometer (Roche, ACCU-CHEK) on blood from tail snip. For intraperitoneal glucose tolerance tests (IPGTT) and oral glucose tolerance tests (OGTT), blood samples from mice fasted for 15 h were collected at 0, 15, 30, 60, and 120 min after an *i.p.* injection of glucose (2 mg/g body weight) or intragastric administration (1 mg/g body weight). For in vivo glucose-stimulated insulin secretion (GSIS), the insulin was measured from serum collected at 0 and 15 min after an *i.p*. injection of glucose (2 mg/g body weight). Insulin concentrations were determined using a radio immune assay (RIA) kit by the manufacturer (Beijing Victor Biotechnology Co., Ltd., RK-146). For insulin tolerance tests, mice fasted for 4 h and were *i.p.* injected with insulin (Beyotime, P3375), following blood glucose measurements at 0, 15, 30, and 60 min.

In omics and histology studies, the serum, pancreas, and liver were collected in fed status for sample preparation or 4% paraformaldehyde (PFA) fixation and paraffin embedding. Major blood biochemical parameters of serum samples were determined with an automatic biochemistry analyzer (Sysmex, BX3010).

### Assessments of energy budget in animals

For energy budget analysis, mice were placed into each Metabolic PhenoCage (TSE Systems) loaded with feed and drinking water in advance. Consumptions of feed, drinking water, and oxygen, CO_2_ production, and other parameters were tracked (about every 20 min). The monitoring data from the 4th day was gathered for statistics.

### Apoptosis and cell viability assays

For the determination of apoptosis in the paraffin-embedded pancreatic section, a TUNEL assay (Beyotime, C1088) was performed according to the manufacturer’s instructions. Images were captured and TUNEL^+^ nuclei surrounded by insulin^+^ staining were determined with the Automated Quantitative Pathology Imaging System and inForm Software (Vectra Polaris).

For determination of apoptosis in PA/rMSA-treated MIN6 cells, an Annexin V-FITC/PI apoptosis detection kit (Mei5bio, MF124) and a GreenNuc™ Caspase-3 Assay Kit for Live Cells (Beyotime, C1168) were used following manufacturers’ instructions. The apoptosis level of cells was analyzed with a flow cytometer (BD, Aria II).

For determination of cell viability, 5,000 MIN6 cells per well were seeded in a 96­well plate and subjected to specific treatments. Cell viability was determined with a Cell Counting Kit-8 (CCK-8; Mei5bio, MF128) according to the manufacturer’s instructions.

### H&E staining and immunofluorescence analyses

Paraffin-embedded tissues were sectioned at 5 μm thickness. Paraffin sections with the largest tissue surface area were used. Sections were dewaxed and rehydrated.

For H&E staining, sections were stained with hematoxylin and eosin successively. Whole images from pancreas slices were captured with the Automated Quantitative Pathology Imaging System (Vectra Polaris). Areas of islets, exocrine tissues, other tissues, and image background were quantified with the inForm Software (Vectra Polaris).

For immunofluorescence (IF) staining, BrdU (Beyotime, ST1056) was *i.p.* injected at 1 mg per mouse for five continuous days before mouse sacrifice. After antigen retrieval and blocking, appropriate primary antibodies were incubated overnight at 4 °C, followed by incubation with FITC/TRITC conjugated secondary antibodies (ZSbio). Dilution ratio of primary antibodies used: anti-insulin (1:200; Abcam, ab181547), anti-cleaved caspase-3 (CC3, 1:50; CST, 9661S), and anti-BrdU (1:200; Abcam, ab6326). Secondary antibodies were applied for 1 h at room temperature, and nuclei were counterstained with DAPI. Imaging was performed with Nikon A1 laser scanning confocal microscope and NIS-Elements Software (Nikon) and analyzed with inForm Software (Vectra Polaris) and Image-ProPlus Software.

### Transmission electron microscopy

Cell pellets were fixed with glutaraldehyde followed by en bloc fixation with 2% (vol/vol) uranyl acetate, fixed in osmium tetroxide, dehydrated by alcohol gradient, and then infiltrated with Spurr’s resin. Transmission electron micrographs of these samples were acquired with an HT7800 instrument (HITACHI Japan). For quantitation of caveolae, only distinctly flask-shaped, noncoated vesicles (50–100 nm in diameter) found on the plasma membranes were scored as caveolae. Total caveolae counts were normalized to the unit length of the plasma membrane (10 μm) and measured using Image J software.

### RNA sequencing and data analysis

A total of 6 liver samples from mice treated with saline or rMSA for 9 weeks were randomly selected to explore the chronic effects of rMSA on the hepatic gene expression profile. AML12 hepatocytes were washed twice with warm PBS and starved in serum-free DMEM overnight. The AML12 hepatocytes were respectively treated with vehicle, insulin (10 nM), rMSA (600 μM), or insulin + rMSA for 24 h. The mirVana RNA Isolation Kit was used to isolate total hepatic RNAs (Life Technologies). For the construction of the cDNA library, RNA samples having a RNA integrity number greater than 8.5 were employed, and rRNA was depleted. A BGISEQ 500 platform was used to do the mRNA sequencing (BGI, Hong Kong). Differentially expressed genes (DEGs) were determined with the R package “DESeq2” (v 1.18.0). With an absolute log_2_ fold change (shrunken) ≥ 1 and a Benjamini–Hochberg adjusted *p* < 0.05, a gene was considered as differentially expressed. The Dr. Tom web tool (https://biosys.bgi.com/) was used to visualize the DEGs and enriched pathways. Gene set enrichment analysis (GSEA) was performed to identify significantly enriched pathways in the KEGG databases using the Dr. Tom web tool.

### Mass spectrometry analysis

Proteomic analysis was performed as described in the previous work [24]. Briefly, an equal weight of the pancreas or livers from each mouse was homogenized in an 8 M urea containing protease and phosphatase inhibitor cocktail (Beyotime, P1048). The mixture was centrifuged, and the concentration of protein was determined in the supernatant. Two hundred micrograms of proteins were desalinized and digested with trypsin. Each sample was labeled with a unique tandem mass tag (TMT) (ThermoFisher, 90110) and subjected to LC–MS/MS analyses respectively. Differentially expressed proteins were determined with the R package “limma” (v 3.42.2). With an absolute log_2_ fold change ≥ 1 and an adjusted *p* < 0.05, a protein was considered as differentially expressed. For gene set enrichment analysis (GSEA), GSEA Software v4.3.2 was used according to the mouse hallmark (MH) gene set that was obtained from MSigDB mouse collections [25, 26].

For lipidomics analysis, MIN6 cells in 6 cm dishes of 80% confluence were treated with 0/0.2 mM PA and 0/300 μM rMSA for 16 h. Cells were harvested, counted, and lysed with 300 μL methanol. Lipids were extracted by adding 600 μL dichloromethane to the mixture and thorough vortex. The organic phase was placed under a nitrogen blower at room temperature until the solvent volatilized, and then redissolved by an equal number of cells. LC–MS/MS was performed as previously described [27, 28].

For the Flux method, AML12 hepatocytes in 6 cm dishes were treated with 0/10 nM insulin and 0/600 μM rMSA for 6 h. After the treatments, AML12 hepatocytes were lysed with 3 ml 80% methanol. The lysate was centrifuged at 12,000×*g* at 4 °C for 20 min. The supernatant was spin-dried and dissolved for LC–MS/MS analyses.

### Glucose uptake assay

AML12 hepatocytes were washed twice with warm PBS and starved in serum-free DMEM (without glucose) overnight. After the pretreatment with vehicle or inhibitors for 30 min, indicated concentrations of rMSA with or without insulin were added. After the treatment, all culture medium was removed from each well and the glucose uptake was detected using a Cell Meter™ 2-NBDG Glucose Uptake Assay Kit (AAT Bioquest, 23500) following the manufacturer’s instruction.

### Fatty acid uptake analysis

To examine the effects of rMSA on fatty acid (FA) uptake using flow cytometry, MIN6 cells cultured in 6-well plates of 80% confluence were treated for 30 min at 37 °C with serum-free RPMI-1640 medium containing rMSA of specific concentrations and 15 μM BODIPY™ FL-C12 to simulate the uptake and metabolism of PA [29].

For FA uptake rate assessments, MIN6 cells were incubated with serum-free RPMI-1640 medium containing 0.2 mM PA and rMSA of specific concentrations. Media at 0, 0.5, 1, 2, 4, 8, 12, and 24 h time points were collected to determine the concentration of FA using an FFA Quantitative Detection Kit (Boxbio, AKFA008M) following the manufacturer’s instruction.

### Western blot analysis

Cells subjected to specific treatments were harvested and lysed in lysis buffer for western blot (Beyotime, P0013) containing protease inhibitor cocktail and phosphatase inhibitor cocktail (Beyotime, P1048). Protein extracts were resolved on an SDS-PAGE gel and transferred to a PVDF membrane. Membranes were incubated overnight at 4 °C with appropriate primary antibodies. Dilution ratio of primary antibodies used: anti-p-AKT (1:1000; CST, 4058S), anti-AKT (1:1000; CST, 9272S), anti-p-GSK3β (1:1000; Proteintech, 67558-1-Ig), anti-GSK3β (1:1000; Proteintech, 22104-1-AP), anti-p-FOXO1/FOXO3a (1:1000; Beyotime, AF605), anti-FOXO1 (1:1000; Beyotime, AF603), anti-β-actin (1:1000; ZSbio, TA-09), anti-p-IGF-1Rβ/IRβ (1:1000; CST, 3024T), anti-IGF-1Rβ (1:1000; CST, 9750T), anti-p-SRC (1:1000; CST, 6943S), anti-SRC (1:1000; CST, 2109S), anti-p-EGFR (1:1000; CST, 2234S), anti-EGFR (1:1000; CST, 4267S), anti-CAV1 (1:1000; CST, 3267S), anti-CC3 (1:1000; CST, 9661S), anti-CHOP (1:1000; Proteintech, 15204-1-AP), anti-p-PERK (1:1000; CST, 3179S), anti-PERK (1:1000; CST, 3192S), anti-β-Tubulin (1:2000; ZSbio, TA-10). Detection of proteins was carried out with a CCD scanner (Tanon, 4600SF) by incubations with horseradish peroxidase (HRP) conjugated secondary antibodies (CST) followed by enhanced chemiluminescence detection reagents (ThermoFisher, 34095).

### Phospho-antibody array analysis

For comparison of the activation and expression of phosphoproteins by rMSA treatment, AML12 hepatocytes were treated with or without rMSA (600 μM) for 24 h. Then, the samples were treated with cell lysis buffer (Full Moon BioSystems, USA). Next, phosphatase inhibitor and protease inhibitor were added to each sample at a volume ratio of 1:50, and each sample was treated with a tube of Full Moon cell lysis beads. After the above steps, the proteins of the samples were extracted, and then, Phospho Explorer Antibody Array (Full Moon BioSystems, USA) was used to react with the protein according to the standard procedures provided by Full Moon. The array contained 1318 antibodies, and each of them had 2 replicates. Wayen Biotechnologies (Shanghai) performed the experiment and analyzed the data. The extent of protein phosphorylation was calculated by the following equation:

$$ {\text{phosphorylation}}\;{\text{ratio}} = {\text{phosphorylated}}\;{\text{value}}/{\text{unphosphorylated}}\;{\text{value}}. $$

### Co-immunoprecipitation

Co-immunoprecipitation was performed using the Immunoprecipitation Kit with Protein A+G Magnetic Beads (Beyotime, P2179S) following the manufacturer’s instruction. The pre-cleared cell lysate was incubated with CAV1 antibody (1:200; CST, 3267S) immobilized on magnetic beads overnight at 4 °C, after which the beads were washed extensively with lysis buffer, eluted, and solubilized in SDS sample loading buffer.

### Glycolysis stress tests

Five thousand AML-12 hepatocytes were seeded on a Seahorse 96-well tissue culture plate (Agilent Technologies). The plate was stood for 30 min for the cells to settle before placing it in the incubator overnight. The adhered cells were treated with or without 600 μM for 6 h before Seahorse analysis. The cells were switched to serum-free Seahorse media before the assay according to the manufacturer’s instructions. For cells subjected to Seahorse XF Glycolysis Stress Test Kit (103020-100, Agilent Technologies), glucose (20 mM), oligomycin (2 mM), and 2-DG (50 mM) were injected sequentially. At the beginning of the assay, the medium was changed to unbuffered, glucose-free DMEM (Sigma-Aldrich Cat D5030, pH 7.35 at 37 °C) supplemented with 2 mM glutamine.

### Statistical analysis

Unless otherwise stated, data are presented as mean ± s.e.m. Data are derived from experiments repeated at least three times unless stated otherwise. Animal studies were performed with the blinding of the experiment operator. If not mentioned otherwise in the figure legend, statistical significance is indicated by *p < 0.05, **p < 0.01, ***p < 0.001, and ****p < 0.0001. Statistical analysis was carried out using unpaired, two­tailed t­test or two­way ANOVA unless otherwise noted. GraphPad Prism 8 and R 4.2.2 were used for statistical analysis.

## Results

### Therapeutic administration of rMSA improves T2DM in mice

**Fig. 1:**
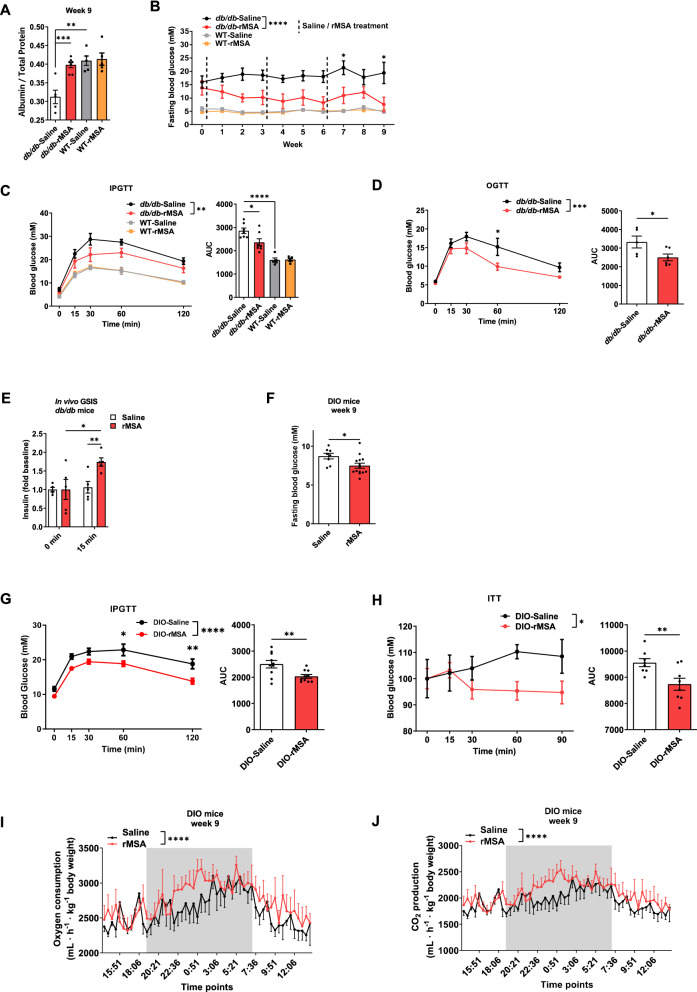
Therapeutic administration of rMSA improved glucose metabolism and insulin secretion in T2DM mice. **A** Serum albumin/total protein levels of *db*/*db* mice (n = 5–7 per group) and WT mice (n = 5 per group). The WT mice and *db*/*db* mice were treated with saline or rMSA for 9 weeks. **B** Fasting blood glucose levels at indicated time points of the WT mice (n = 5 per group) and hyperglycemic *db*/*db* mice (n = 6 per group) respectively treated with saline or rMSA for 9 weeks. **C** Intraperitoneal glucose tolerance test (IPGTT) and area under the curves (AUC) in the WT and *db*/*db* mice (n = 5–7 per group). The WT mice and *db*/*db* mice were treated with saline or rMSA for 6 weeks. **D** Oral glucose tolerance test (OGTT) and corresponding AUC in the *db*/*db* mice (n = 5 per group). The *db*/*db* mice were treated with saline or rMSA for 6 weeks. **E** Glucose-stimulated insulin secretion (GSIS) assay in the *db*/*db* mice treated with saline or rMSA for 6 weeks at 0 or 15 min (n = 5 per group). **F** Fasting blood glucose levels of DIO mice (n = 8–14 per group). The DIO mice were treated with saline or rMSA for 9 weeks. **G** IPGTT and corresponding AUC in DIO mice (n = 10 per group). The DIO mice were treated with saline or rMSA for 9 weeks. **H** Insulin tolerance test (ITT) and corresponding AUC in DIO mice (n = 8 per group). The DIO mice were treated with saline or rMSA for 9 weeks. **I** Oxygen consumption of the DIO mice (n = 4 per group) treated with saline or rMSA for 9 weeks. The region marked in grey represents the nighttime. **J** CO2 production of DIO mice (n = 4 per group) treated with saline or rMSA for 9 weeks. The region marked in grey represents the nighttime. Data were analyzed by two-way ANOVA with repeated measures (**B**–**D** and **G**–**J**) and unpaired t-tests (**A** and **C**–**H**). Data are expressed as mean ± s.e.m. *p < 0.05, **p < 0.01, ***p < 0.001, ****p < 0.0001

To verify the clinical observation of reduced serum albumin levels in T2DM patients [14, 15], *db*/*db* mice and WT mice were respectively treated with young and undamaged rMSA or saline to compare serum albumin and total protein levels after treatments. The young and undamaged rMSA used in this study was the same as that in our previous study, and the long-time administration of the rMSA was found to be safe and to have the ability to extend the lifespan of aging mice [22]. We found that the level of serum albumin, total proteins, as well as the ratio of albumin over total proteins were significantly up-regulated in rMSA-treated *db*/*db* mice (Fig. 1A, Additional file 1: Fig. S1A, B). In line with our prior findings, rMSA treatment had no effect on serum albumin or total protein levels in WT mice [22]. To investigate whether restoring the serum albumin level can improve the blood glucose homeostasis, *db*/*db* mice were injected with rMSA or saline. The results showed that the fasting blood glucose level (FBGL) was significantly reduced in rMSA-treated *db*/*db* mice (Fig. 1B) and neither rMSA nor saline affected the FBGL of the WT mice (Fig. 1B). Furthermore, we found that the rMSA-treated *db*/*db* mice had better intraperitoneal glucose tolerance tests (IPGTT) performance (Fig. 1C). Since the incretin effect triggered by oral glucose can significantly enhance glucose-mediated insulin release [30], we also performed oral glucose tolerance tests (OGTT) in *db*/*db* mice and found that rMSA significantly improved the OGTT of *db*/*db* mice (Fig. 1D). At the same time, in vivo glucose stimulated insulin secretion (GSIS) showed that the islets of rMSA-treated *db*/*db* mice could better respond to glucose stimulation and secrete insulin (Fig. 1E).

To verify the above observations, diet-induced obesity (DIO) mice were employed for rMSA or saline treatment. We found that the level of serum albumin was significantly up-regulated in rMSA-treated DIO mice (Additional file 1: Fig. S1C). The rMSA-treated DIO mice had a significantly lower FBGL, a better IPGTT, and a better insulin tolerance tests (ITT) performance than the saline-treated DIO mice (Fig. 1F–H). In addition, we found that the rMSA treatments reduced the body weight of DIO mice and had no effect on the food intake (Additional file 1: Fig. S1D, E). The oxygen consumption and CO_2_ production of rMSA-treated mice were both increased (Fig. 1I, J), indicating that the overall metabolism level was higher. In summary, these data show that pharmacological administration of rMSA can alleviate T2DM in mice.

### rMSA prevents islet atrophy and β-cell apoptosis

**Fig. 2:**
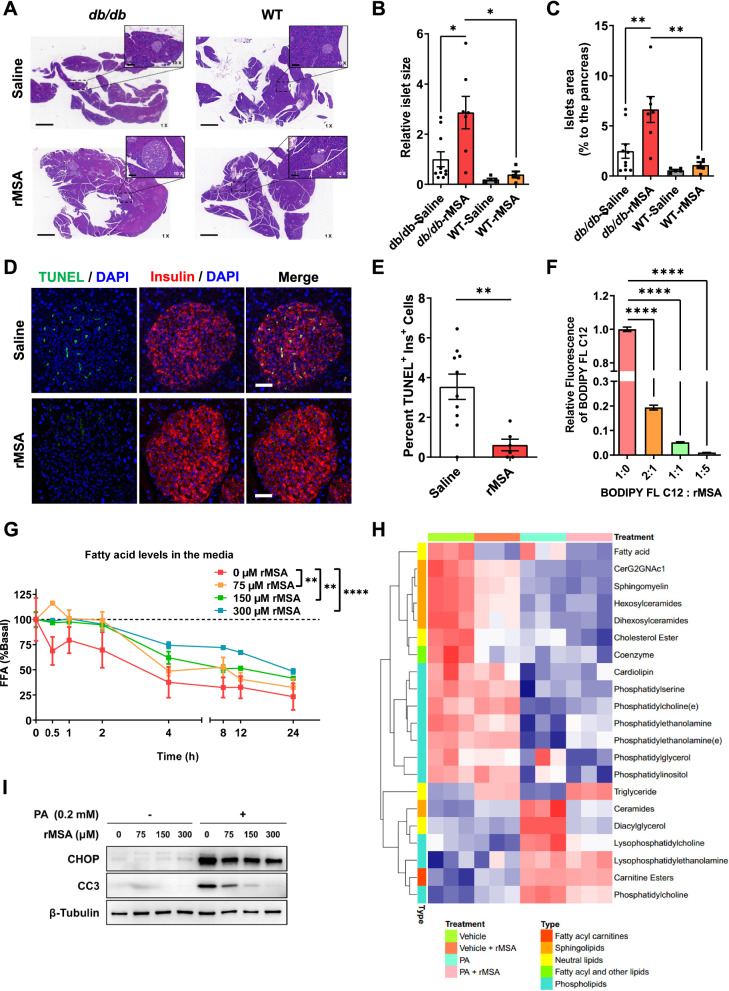
rMSA prevented islet atrophy and β-cell apoptosis by reducing FFA uptake. **A**–**C** Representative images of H&E staining of pancreatic slices from the *db/db* mice (n = 7–10 per group) and WT mice (n = 5 per group) respectively treated with saline or rMSA for 9 weeks (**A**). Dotted boxes indicate the location of the islets, with the corresponding ×10 zoom in the upper right corner of each ×1 image. Scale bars in the ×1 images represent 1 mm, and those in the ×10 images represent 100 μm. The relative size (**B**) and proportion of islets to the pancreas (**C**) were calculated respectively. **D** Representative images of IF staining for insulin and TUNEL assays in pancreases of the *db*/*db* mice treated with saline (n = 10) or rMSA (n = 6) for 9 weeks. Insulin staining is shown in red; DAPI-stained nuclei are shown in blue; TUNEL is shown in green. Scale bars represent 50 μm. **E** The percentage of TUNEL and insulin double-positive cells to all insulin-positive cells in the islets of the *db*/*db* mice treated with saline or rMSA for 9 weeks, corresponding to the representative images (**D**). **F** Flow cytometry analysis showing the relative fluorescent intensity in MIN6 cells under the indicated molar ratios of BODIPY™ FL C12 to rMSA (n = 3 per group). **G** FFA uptake assays showed the effects of rMSA at different concentrations (n = 3 per group) on FFA uptake rate, which was represented as the concentration of FFA in the medium at different time points. The basal concentration of FFA under each treatment was 0.2 mM. **H** Heat map showing the PA (0 or 0.2 mM) and rMSA (0 or 300 μM) treatments for 16 h on the intracellular lipidome of MIN6 cells (n = 3 per group). **I** Western blot showing the protein levels of CC3 and CHOP in the MIN6 cells treated with vehicle or PA (0.2 mM) with rMSA at indicated concentrations. β-Tubulin was used as the internal reference. Data were analyzed by two-way ANOVA with repeated measures (**G**) and unpaired t-tests (**B**, **C**, **E**, and **F**). Data are expressed as mean ± s.e.m. *p < 0.05, **p < 0.01, ****p < 0.0001

Based on the above observations, we wondered whether the rMSA treatments improved the function of islet β-cells in T2DM mice. As we expected, besides the GSIS (Fig. 1E), the fed blood insulin level was found to be significantly improved in rMSA-treated *db*/*db* mice (Additional file 1: Fig. S2A). The quantitative proteomic analysis on the pancreas showed that the protein levels of insulin-1 and insulin-2 were up-regulated in the rMSA group (Additional file 1: Fig. S2B–D). Meanwhile, gene set enrichment analysis (GSEA) of the proteomic data showed that the gene set of PANCREAS_BETA_CELLS was significantly enriched in the rMSA group (Additional file 1: Fig. S2E), indicating the pancreas from rMSA-treated *db*/*db* mice had higher β-cell-related features. More importantly, histological observations showed that the size of islets from rMSA-treated *db*/*db* mice was significantly larger than that of the saline-treated mice (Fig. 2A, B), and the proportion of the islet area to the entire pancreatic section was significantly higher than that of the saline group (Fig. 2A, C). For WT mice, the rMSA treatments had no obvious effect on the islet size and proportion, although the islet compensation occurred in the rMSA-treated *db*/*db* mice (Fig. 2A–C). Since it was reported that islet β-cell compensation and apoptosis determines the size of islets at different stages of T2DM [31, 32], we hypothesized that in *db*/*db* mice the saline group had a higher degree of islet atrophy and the rMSA was unlikely to cause further islet β-cell proliferation. As expected, the TUNEL assay showed that the percentage of TUNEL^+^ β-cells in islets of the saline group was significantly higher than that in the rMSA group (Fig. 2D, E). Consistently, the number of CC3^+^ β-cells in saline-treated mice was also significantly higher than those in the rMSA group (Additional file 1: Fig. S3A, B). Regarding the compensatory proliferation, the percentage of BrdU^+^ β-cells analyzed by IF staining showed no significant difference in the two groups (Additional file 1: Fig. S3C, D). In sum, the saline group had a smaller islet size due to a higher degree of islet β-cell apoptosis.

It was reported that lipotoxicity mediated by FFA over-uptake can cause apoptosis in islet β-cells [8]. GSEA of the proteomics data from the pancreas of the *db*/*db* mice showed that the gene set of FATTY_ACID_METABOLISM was significantly enriched in the saline-treated mice (Additional file 1: Fig. S2E, F), which suggested that the saline-treated mice had higher protein levels of these genes to metabolize the fatty acid over-uptaken. Furthermore, we investigated protein levels associated with fatty acid catabolism (GO: 0009062). It was found that 14 proteins had lower levels in the pancreas of the rMSA-treated mice (Additional file 1: Fig. S2G). Although rMSA injection did not influence FFA levels in the serum (Additional file 1: Fig. S4A), and there is no clear report about the link between β-cell apoptosis and the FFA/albumin molar ratios. We thus hypothesized that the β-cell protection by rMSA was reducing lipid uptake by islet β-cells through decreasing the FFA/albumin molar ratio, which was exactly what we found here (Additional file 1: Fig. S4B). Hence, we determined the uptake of BODIPY™ FL C12 fluorescent probe (to mimic PA) by MIN6 cells under different molar ratios. The results showed that with the decrease of the molar ratio, the uptake of fluorescent probes by MIN6 cells decreased (Fig. 2F, Additional file 1: Fig. S4C). Furthermore, FFA uptake assays showed that, when there was no rMSA, the concentration of FFA decreased significantly within 30 min, and decreased to about 25% of the basal concentration in the medium within 24 h (Fig. 2G). In the presence of rMSA, FFA concentrations in the media began to decrease after 2 h. When there was 300 μM rMSA, FFA concentration decreased to 50% within 24 h (Fig. 2G). These findings demonstrated that rMSA could significantly slow down the uptake of FFA by MIN6 cells dose-dependently.

Accumulated ceramide caused by FFA over-uptake is an important substance in lipotoxic-mediated ER stress of islet β-cells that can lead to cell apoptosis [9]. The lipidomic analysis showed that, without rMSA, PA treatments led to an obvious elevation of ceramide level, which was reversed when rMSA was added to the system (Fig. 2H, Additional file 1: Fig. S4D). To verify the effects of the changes in lipidome on ER stress and apoptosis, CHOP and CC3 levels were thus measured after PA and rMSA treatments for 16 h, which showed that PA treatments increased levels of CHOP and CC3, whereas rMSA reversed these effects dose-dependently (Fig. 2I). Furthermore, the relative viability of MIN6 cells treated with PA and rMSA for 16 h was determined, which showed that PA treatments could significantly impair the cell viability; with the increase of rMSA concentration, the impairment was alleviated (Additional file 1: Fig. S5A). Similarly, apoptosis assays showed that PA treatments for 16 h significantly induced apoptosis and increased caspase-3 activity of MIN6 cells (Additional file 1: Fig. S5B–H). With the increase in rMSA concentration, the number of apoptotic and caspase-3 activated cells decreased significantly (Additional file 1: Fig. S5B–H). It is consistent with the hypothesis that rMSA alleviates ER stress and apoptosis in islet β-cells by reducing lipid uptake and relieving ceramide accumulation. Therefore, a series of inhibitors targeting key nodes in these processes were used to verify the role of these processes in lipotoxicity-induced islet β-cell apoptosis. Firstly, sulfo-*N*-succinimidyl oleate sodium (SSO), an effective inhibitor of fatty acid transporter CD36 [33], was found to reduce the levels of CC3 and CHOP induced by PA (Additional file 1: Fig. S6A). Secondly, fumonisin B1 was used to block ceramide de novo synthesis [34] and was observed to reduce the phosphorylation of PERK and the level of CC3 in PA-treated MIN6 cells (Additional file 1: Fig. S6B). Thirdly, the phosphorylation of PERK, another key node of ER stress besides CHOP, was thought to be engaged in PA-induced MIN6 apoptosis. It was found that the PERK antagonist GSK2656157 reduced levels of p-PERK, CHOP, and CC3 (Additional file 1: Fig. S6B). Correspondingly, compared with PA, the PERK agonist CCT020312 increased levels of p-PERK, CHOP, and CC3, but rMSA failed to reduce this effect (Additional file 1: Fig. S6C). In addition, although it was found that rMSA could be highly internalized by MIN6 cells as analyzed by flow cytometry (Additional file 1: Fig. S6D), it is not clear whether the anti-apoptosis function of rMSA depends on direct contact with islet β-cells. To answer this question, MIN6 cells were exposed to PA and co-incubated with the immobilized rMSA without direct contact with the cells for 16 h. The results showed that both the immobilized rMSA and free-rMSA could reduce the PA-induced CC3 level (Additional file 1: Fig. S6E). Taken together, we demonstrated that the PA over-uptake induces ceramide accumulation and then induced ER stress and apoptosis in islet β-cells, which can be dose-dependently reversed by rMSA without direct contact with the cells.

### rMSA increases glycolysis in hepatocytes

**Fig. 3:**
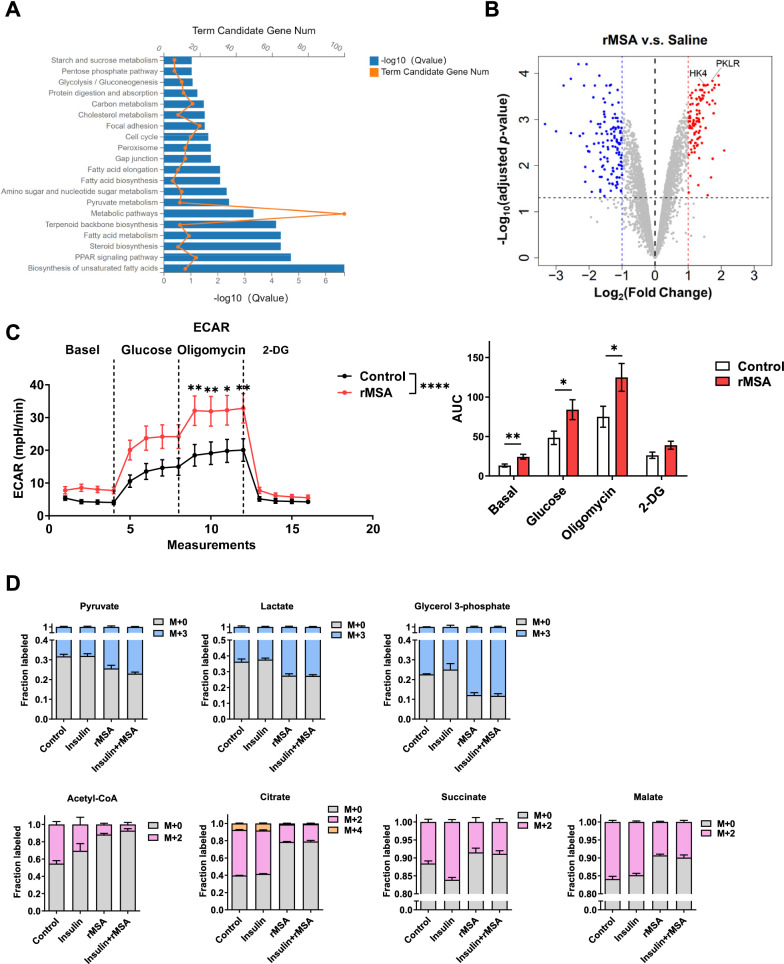
rMSA increased glycolysis and upregulated glycolysis-related proteins in hepatocytes. **A** Pathway enrichment analysis of upregulated genes in the livers of rMSA-treated *db*/*db* mice compared with saline-treated *db*/*db* mice for 9 weeks (n = 3 per group). **B** Volcanic map showing the protein levels identified by quantitative mass spectrometry in the livers of *db*/*db* mice (n = 3 per group) treated with saline or rMSA for 9 weeks. The blue and red dots on the outside of the blue and red vertical dashed lines respectively show proteins with down-regulation and up-regulation by 2 times with an adjusted *p* < 0.05 (above the gray horizontal dashed line) in the rMSA group compared with the saline control. The two remarkably up-regulated proteins (HK4 and PKLR) are marked. **C** Metabolic profile on AML12 hepatocytes (n = 11 per group) with or without rMSA (600 μM) treatments for 6 h as measured by Seahorse glycolytic stress assay. The corresponding AUC of each treatment is shown. **D** Isotope tracing of [U-13C]-glucose metabolism in AML12 hepatocytes (n = 3 per group) treated with vehicle, insulin (10 nM, 6 h), rMSA (600 μM, 6 h), and insulin (10 nM, 6 h) + rMSA (600 μM, 6 h). Data were analyzed by two-way ANOVA with repeated measures (**C**) and unpaired t-tests (**C**). Data are expressed as mean ± s.e.m. *p < 0.05, **p < 0.01, ****p < 0.0001

The above mechanism of rMSA protecting islet β-cells from lipotoxicity-induced apoptosis can be explained by the classical function of albumin, namely the transport and regulation of fatty acid uptake. Meanwhile, we found that rMSA has a novel function on the liver for T2DM improvement. Since the liver is an essential organ of glucose metabolism [35], we isolated and subjected the liver samples from *db*/*db* mice treated with saline or rMSA to transcriptomic and proteomic analyses. Functional annotation and enrichment analysis of the differentially expressed genes (DEGs) showed that a variety of metabolic processes were up-regulated by rMSA treatments (Fig. 3A, Additional file 1: Fig. S7A). Proteomic analyses revealed that hexokinase 4 (HK4) and pyruvate kinase isozymes R/L (PKLR), which are the rate-limiting enzymes of glycolysis, were significantly up-regulated by rMSA treatments (Fig. 3B). Based on these findings, glycolysis stress tests on AML12 hepatocytes showed that extracellular acidification rates (ECAR) and oxygen consumption rates (OCR) were increased by rMSA treatments (Fig. 3C, Additional file 1: Fig. S7B), suggesting that the glycolysis was enhanced. Since insulin plays an essential role in the regulation of hepatic glucose metabolism [36], the [U-^13^C] glucose was used to assess labeled metabolites in vehicle-, insulin-, rMSA-, and insulin + rMSA-treated AML12 hepatocytes, respectively. It was observed that the rMSA treatments significantly increased the ratio of isotope-labeled glycolytic products (Fig. 3D). Similarly, the relative content of glycolytic products and intermediates also increased (Additional file 1: Fig. S7C). Taken together, the treatments of rMSA in AML12 hepatocytes increased the incorporation of glucose into glycolysis and promoted glycolysis.

### rMSA promotes the PI3K-AKT signaling via EGFR

**Fig. 4:**
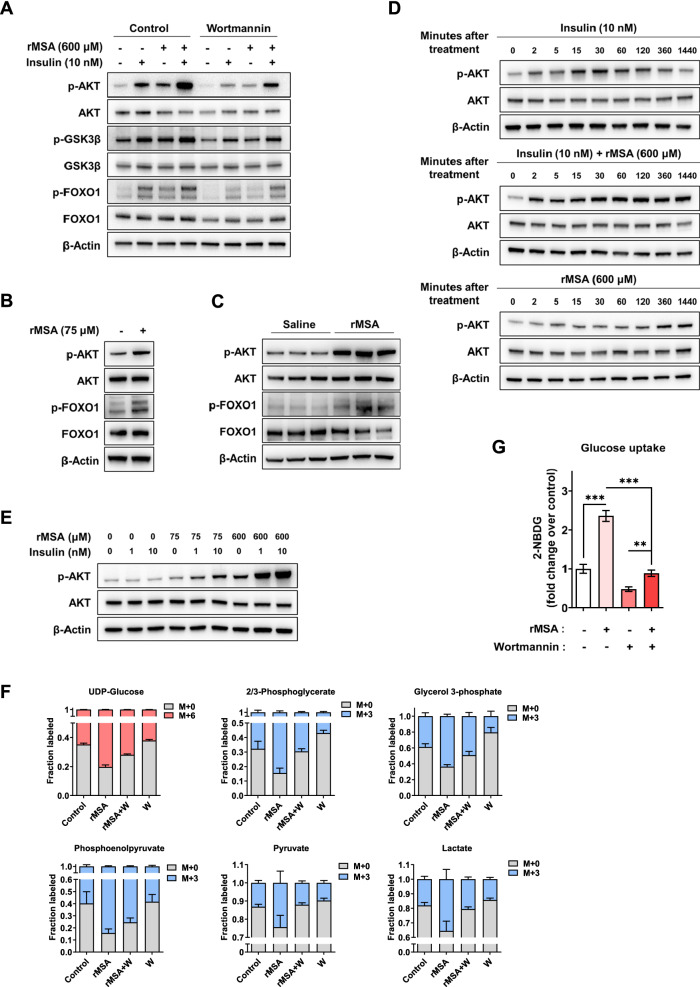
rMSA increased glycolysis and glucose uptake by upregulating the PI3K-AKT signaling in hepatocytes. **A** Western blot showing the protein and phosphorylation levels of AKT, GSK3β, and FOXO1 in the AML12 hepatocytes treated with vehicle or rMSA (600 μM), with or without insulin (10 nM) in the absence or presence of 1 μM PI3K inhibitors Wortmannin for 6 h. **B** Western blot showing the protein and phosphorylation levels of AKT and FOXO1 in the primary hepatocytes treated with vehicle or rMSA (75 μM) for 24 h. **C** Western blot showing the protein and phosphorylation levels of AKT and FOXO1 in the livers of db/db mice (n = 3 per group) treated with saline or rMSA for 9 weeks. **D** Western blot showing the protein and phosphorylation levels of AKT in the AML12 hepatocytes treated with vehicle or rMSA (600 μM), with or without insulin (10 nM) at different time points. **E** Western blot showing the protein and phosphorylation levels of AKT in the AML12 hepatocytes treated with vehicle or rMSA (75, 600 μM), with or without insulin (1, 10 nM) for 24 h. **F** Isotope tracing of [U-13C]-glucose metabolism in AML12 hepatocytes (n = 3 per group) treated with vehicle or rMSA (600 μM, 6 h) in the absence or presence of 1 μM Wortmannin (W). **G** 2-NBDG uptake in AML12 hepatocytes (n = 3 per group) treated with vehicle or rMSA (600 μM, 6 h) in the absence or presence of 1 μM Wortmannin. Data were analyzed by unpaired t-tests (**G**). Data are expressed as mean ± s.e.m. **p < 0.01, ***p < 0.001. β-Actin was used as the internal reference (**A**–**E**)

Based on the observations of the promoted glycolysis by rMSA treatments, we wondered which intracellular signaling pathways were enhanced when hepatocytes were exposed to rMSA. The AML12 hepatocytes were respectively treated with vehicle, insulin, rMSA, or insulin + rMSA for 24 h and subjected to RNA-seq analyses. Both “rMSA vs. vehicle” and “rMSA + insulin vs. insulin” were compared, and 246 DEGs were selected (Additional file 1: Fig. S8A) to perform the pathway enrichment analysis. Results showed that these 246 DEGs were mainly enriched in TNF signaling pathway, IL-17 signaling pathway, and PI3K-AKT signaling pathway (Additional file 1: Fig. S8B). Certainly, the PI3K-AKT pathway has been reported to play a key role in regulating metabolism in response to growth factor activation [4]. To interrogate the impacts of rMSA on the PI3K-AKT signaling pathway in detail, the AML12 hepatocytes were treated with insulin, rMSA, or insulin together with rMSA, respectively, with or without PI3K inhibitors wortmannin, PI-103, or ZSTK474 as indicated. The data showed that insulin, rMSA, or insulin together with rMSA can all enhance the phosphorylation of AKT, which could be attenuated by PI3K inhibitors (Fig. 4A, Additional file 1: Fig. S8C). And the activation of GSK3β and FOXO1, which were reported to be related to glucose metabolism [4], could also be eliminated by wortmannin, suggesting that the metabolic changes of AML12 hepatocytes caused by rMSA treatment are regulated by the PI3K-AKT signaling (Fig. 4A). Consistently, we found that rMSA treatment increased the phosphorylation of AKT and FOXO1 in mouse primary hepatocytes (Fig. 4B). In addition, the phosphorylation levels of AKT and FOXO1 in the livers from rMSA-treated *db*/*db* mice were up-regulated (Fig. 4C). Notably, there was a strong synergistic effect between rMSA and insulin (Fig. 4A). Experiments on time course and concentration gradients showed that rMSA could significantly activate AKT and prolong the effect of insulin on AKT activation (Fig. 4D, E).

To demonstrate the role of PI3K-AKT signaling in rMSA-enhanced glycolysis, [U-^13^C] glucose was used to assess labeled metabolites in vehicle-, rMSA-, wortmannin (W)-, and rMSA + wortmannin (W)-treated AML12 hepatocytes, respectively. The increased proportion and relative content of glycolysis-related compounds caused by rMSA treatments were reversed by wortmannin (Fig. 4F, Additional file 1: Fig. S8D). Because of the high glycolysis flux yields in hepatocytes [37], labeling results were confirmed by glucose uptake measurements. As demonstrated by the complete blockade of rMSA-increased glucose uptake in the presence of Wortmannin in AML12 hepatocytes, PI3K-AKT was found to be required (Fig. 4G). Taken together, these results confirm that rMSA promotes the PI3K-AKT pathway activation to enhance glycolysis and glucose uptake in hepatocytes.

**Fig. 5:**
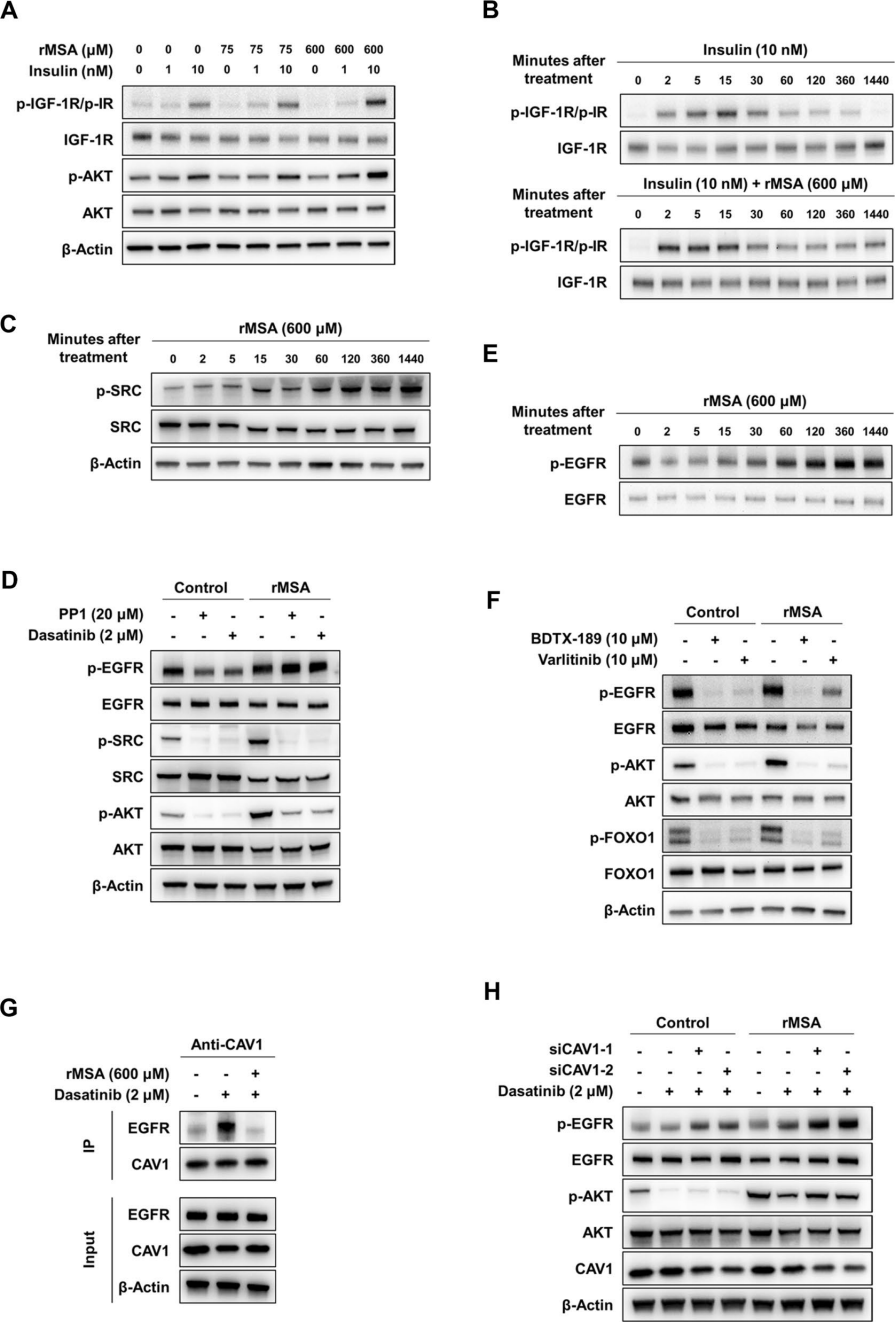
rMSA activated SRC and reduced the interaction of CAV1 between EGFR to increase EGFR activation. **A** Western blot showing the protein and phosphorylation levels of IGF-1R/IR and AKT in the AML12 hepatocytes treated with vehicle or rMSA (75, 600 μM), with or without insulin (1, 10 nM) for 5 min. **B** Western blot showing the protein and phosphorylation levels of IGF-1R/IR in the AML12 hepatocytes treated with vehicle or rMSA (600 μM), with or without insulin (10 nM) at different time points. **C** Western blot showing the protein and phosphorylation levels of SRC in the AML12 hepatocytes treated with rMSA (600 μM) at different time points. **D** Western blot showing the protein and phosphorylation levels of EGFR, SRC, and AKT in the AML12 hepatocytes treated with vehicle or rMSA (600 μM) in the absence or presence of 20 μM PP1 or 2 μM Dasatinib for 2 h. **E** Western blot showing the protein and phosphorylation levels of EGFR in the AML12 hepatocytes treated with rMSA (600 μM) at different time points. **F** Western blot showing the protein and phosphorylation levels of EGFR, AKT, and FOXO1 in the AML12 hepatocytes treated with vehicle or rMSA (600 μM) in the absence or presence of 10 μM BDTX-189 or 10 μM Varlitinib for 6 h. **G** The AML-12 hepatocytes treated with or without rMSA (600 μM) for 2 h in the absence or presence of 2 μM Dasatinib were lysed; comparable amounts of total cell lysates immunoprecipitated with anti-CAV1 antibody were resolved by SDS/PAGE and visualized by the indicated antibodies. **H** Western blot showing the protein and phosphorylation levels of EGFR, AKT, and CAV1 in the AML12 hepatocytes transfected with siRNA against CAV1 (siCAV1-1 and siCAV1-2) for 48 h then treated with vehicle or rMSA (600 μM) in the absence or presence of 2 μM Dasatinib for 2 h. β-Actin was used as the internal reference (**A**, **C**, **D**, and **F**–**H**)

Because the rMSA-induced signaling pathway might resemble that of insulin, we were curious about whether rMSA directly engages in the insulin or insulin-like growth factor 1 receptors (IR and IGF-1R, respectively), or acutely sensitizes hepatocytes to insulin. To test these possibilities, we treated AML12 hepatocytes with insulin for 5 min in the presence or absence of rMSA, respectively. We observed that rMSA did not activate AKT, IR, or IGF-1R at 5 min, but there was an additional effect of the rMSA and insulin co-treatment over insulin alone on AKT, IR, and IGF-1R phosphorylation (Fig. 5A). In addition, experiments on time course showed that rMSA could enhance and prolong the effect of insulin on IGF-1R or IR activation (Fig. 5B). These results revealed that rMSA has a potentiation on insulin. To address whether the presence of intact insulin receptors is required for rMSA-induced AKT phosphorylation, the dual receptor tyrosine kinase inhibitor OSI-906 was used to specifically target IR and IGF-1R. As expected, insulin signaling was completely abolished in the presence of 1 μM OSI-906 but rMSA can still induce AKT phosphorylation (Additional file 1: Fig. S9A, B). Similarly, insulin-induced glucose uptake was significantly reduced with OSI-906, while rMSA-induced glucose uptake was not affected (Additional file 1: Fig. S9C). These results suggested that there is at least another parallel pathway, in addition to the insulin pathway, for rMSA to activate PI3K-AKT signaling.

To explore the pathway by which rMSA enhanced PI3K-AKT signaling independently of insulin receptors, we performed phosphorylated antibody microarray and enrichment analysis in AML12 hepatocytes. Results showed that the ERBB signaling pathway was significantly up-regulated by rMSA treatments (Additional file 1: Fig. S9D). It has been reported that the phosphorylation of SRC can enhance PI3K-AKT signaling activated by growth factors [38]. And studies have shown that albumin can bind GP60 on the plasma membrane to promote the CAV1-mediated caveolae formation, thereby activating SRC signaling and promoting endocytosis [39, 40]. Thus, we examined the SRC, AKT, and EGFR signaling in AML12 hepatocytes. Firstly, experiments on time course showed that rMSA treatments increased the phosphorylation of SRC (Fig. 5C). Secondly, we found that rMSA treatments increased the number of CAV1-mediated caveolae on the plasma membrane (Additional file 1: Fig. S9E). And we also observed that, in the presence of SRC inhibitors (PP1 and dasatinib), the phosphorylation of SRC and AKT were decreased with or without rMSA treatments. (Fig. 5D). These data showed that the activation of AKT by rMSA is partially mediated by SRC. We noticed that the phosphorylation of epidermal growth factor receptor (EGFR) was decreased by SRC inhibitors (Fig. 5D), which is consistent with previous report [41]. Unexpectedly, rMSA treatments reversed this effect (Fig. 5D). Experiments on time course showed that rMSA could increase EGFR phosphorylation (Fig. 5E). We have also observed that the phosphorylations of EGFR, AKT, and FOXO1 were remarkably reduced in the presence of EGFR inhibitors (BDX-189 and Varlitinib) (Fig. 5F). Furthermore, we found that the inhibition of PI3K did not affect the phosphorylation of EGFR (Additional file 1: Fig. S9F), which confirmed that EGFR was the upstream of AKT signaling. Consistently, we found that rMSA treatment increased the phosphorylation of EGFR in mouse primary hepatocytes (Additional file 1: Fig. S9G).

Surely, we want to know the mechanism of increased EGFR phosphorylation by rMSA treatments. Since it is well accepted that the interaction between CAV1 and EGFR prevents the phosphorylation of EGFR [42, 43], we thus suspected that rMSA can reduce the interaction between CAV1 and EGFR. Therefore, we used the CAV1 antibody for immunoprecipitation. We found that, in the presence of the SRC inhibitor, the interaction between EGFR and CAV1 was increased, while the interaction between EGFR and CAV1 was basically abolished by rMSA treatments (Fig. 5G). In addition, we knockdown CAV1 with siRNA in AML12 hepatocytes, and treated the wild type and CAV1-knockdown AML12 hepatocytes with or without the rMSA in the presence of the SRC inhibitor. We found that EGFR phosphorylation increased after the CAV1 knockdown or the rMSA treatment, and there was a synergistic effect between the CAV1 knockdown and the rMSA treatment (Fig. 5H).

In summary, these data show that rMSA treatments can increase SRC phosphorylation and prevent the interaction between CAV1 and EGFR, thereby increasing EGFR phosphorylation thus enhancing PI3K-AKT signaling in hepatocytes.

## Discussion

Human serum albumin (HSA) is clinically used in large doses to treat burns, shock, and blood loss. This study suggested that T2DM can be improved by albumin administration. However, donated human blood is now the sole or primary source of commercially available HSA. As a result, there is a risk of spreading dangerous viruses, triggering allergies, and other adverse reactions [44, 45], which limits the application of albumin in the treatment of chronic diseases such as T2DM. Large-scale manufacturing of recombinant human serum albumin (rHSA) using yeast, bacteria, or plant-based expression systems is on the horizon in order to fulfill the demand for albumin and reduce the danger of the presence of harmful viruses [46]. Hence, attempts to develop recombinant albumin of injection grade to replace blood-derived endogenous albumin are ongoing. The rMSA we have obtained so far has been proven to be not only ultra-pure but also young and undamaged, i.e. intact free-thiol, free of carbonylation, free of AGE and homocysteine modifications [22], which theoretically gives it greater reducibility and the ability to protect other proteins than blood-derived endogenous albumin. In this study, rMSA was found to safely alleviate T2DM by improving hepatic glycolysis through EGFR and protecting islet β cells in mice. Therefore, the development of young, undamaged, and ultrapure rHSA is expected to provide an alternative treatment for human T2DM.

Previous reports have described the activation of PI3K-AKT by albumin in LLC-PK1 cells [47], HKC-8 cells [48, 49], and several other cell lines [50–53]. However, how the PI3K-AKT signaling is activated by albumin is not clear. Recently, albumin endocytosis has been proven to activate the PI3K-AKT pathway, which stimulates albumin endocytosis in the feedforward mechanism [47]. In this study, we revealed novel functions and mechanisms of the rMSA for the metabolic regulations in T2DM mice, which reflected in that rMSA treatments led to an increase in glucose flux and flux-driven glucose uptake, mediated by the activation of PI3K-AKT signaling in hepatocytes. We report here for the first time that rMSA promotes the activation of EGFR by reducing the interaction between CAV1 and EGFR, thereby activating the PI3K-AKT signaling pathway in hepatocytes. CAV1 was initially identified to inhibit EGFR signaling through receptor isolation [42]. However, the formation of caveolae has been shown to promote EGFR signaling [54]. These examples of different roles of CAV1 in EGFR regulation may also indicate the cellular environment dependence of CAV1 function. Our study found that in serum-starved hepatocytes, part of EGFR interacted with CAV1, which was significantly enhanced by the inhibition of SRC signaling. In this condition, rMSA treatments reduced the interaction between EGFR and CAV1 and increased EGFR phosphorylation. Therefore, the present study revealed that albumin acts as an important component of EGFR-PI3K-AKT signaling in hepatocytes.

T2DM can lead to decreased serum albumin levels, and insulin is clinically needed to prevent hypoalbuminemia [11, 55]. To our understanding, it is very important to maintain sufficient albumin levels to alleviate T2DM. In this study, it was observed that rMSA treatments increased the PI3K-AKT signaling in hepatocytes and enhanced the activation of IR/IGF-1R by insulin, and these phenomena were promoted by a higher concentration of rMSA. Therefore, the decreased serum albumin level may impair the insulin sensitivity of hepatocytes in T2DM. Furthermore, the decrease of serum albumin level resulted in the rise of serum FFA/albumin molar ratio, which in turn led to islet β-cell apoptosis caused by excessive lipid uptake. Although we believe that these two effects are the major reasons for the clinically identified decreased albumin levels as a risk factor for T2DM, whether the protective effect of rMSA against lipotoxicity at the cellular level is also applicable to that of in vivo needs to be confirmed in future studies.

In addition to the above mechanisms, it was reported that both ligand binding and anti-oxidant capabilities of HSA are impaired in individuals with diabetes mellitus [56–58]. Patients with T2DM have lower levels of HSA, which may make oxidative stress worse [57]. Furthermore, HSA may prevent other proteins from being glycated in the early stages of diabetes [59]. Therefore, the injection of young and undamaged recombinant albumin may also improve the symptoms of T2DM by rescuing the endogenous albumin, such as relieving oxidative stress and reducing the glycation levels of other proteins.

## Conclusion

In conclusion, for T2DM mice, we found that rMSA treatments increased the serum albumin levels and then improved the blood glucose homeostasis, including glucose tolerance, insulin sensitivity, GSIS, and FBGL. On tissue and cellular levels, the rMSA treatment promoted glucose uptake and glycolysis in hepatocytes by regulating the PI3K-AKT pathway via EGFR, and relieved lipotoxicity-induced β-cell apoptosis and thus ameliorated the islet atrophy. Through these improvements, rMSA treatments prevented T2DM progress in mice.

## Supplementary Information

**Additional file 1: Figure S1.** Therapeutic administration of rMSA increased total protein and serum albumin levels in T2DM mice. **Figure S2.** Therapeutic administration of rMSA increased the insulin level and decreased the fatty acid metabolism in the pancreas of T2DM mice. **Figure S3.** Therapeutic administration of rMSA prevented β-cell apoptosis in T2DM mice. **Figure S4.** rMSA decreased the molar ratio of serum FFA to albumin in T2DM mice and reduced the FFA uptake in islet β- cells. **Figure S5.** rMSA reduced lipotoxicity-induced islet β- cell apoptosis. **Figure S6.** rMSA alleviates ER stress and apoptosis in islet β-cells by reducing lipid uptake. **Figure S7.** rMSA upregulated glycolysis-related gene expression and increased glycolysis in hepatocytes. **Figure S8.** rMSA promoted glycolysis by upregulating the PI3K-AKT pathway in hepatocytes. **Figure S9.** rMSA upregulated the PI3K-AKT pathway through EGFR independently of IR or IGF-1R.

## Data Availability

The datasets during and/or analyzed during the current study available from the corresponding author on reasonable request.
