# Supplementary material for: Young and undamaged recombinant albumin alleviates T2DM by improving hepatic glycolysis through EGFR and protecting islet β cells in mice
Source: J Transl Med. 2023 Feb 6;21:89. doi: 10.1186/s12967-023-03957-3 (PMC9903539; doi:10.1186/s12967-023-03957-3)
Supplement: Supplementary file 1 — Additional file 1: Figure S1. Therapeutic administration of rMSA increased total protein and serum albumin levels in T2DM mice. Figure S2. Therapeutic administration of rMSA increased the insulin level and decreased the fatty acid metabolism in the pancreas of T2DM mice. Figure S3. Therapeutic administration of rMSA prevented β-cell apoptosis in T2DM mice. Figure S4. rMSA decreased the molar ratio of serum FFA to albumin in T2DM mice and reduced the FFA uptake in islet β- cells. Figure S5. rMSA reduced lipotoxicity-induced islet β- cell apoptosis. Figure S6. rMSA alleviates ER stress and apoptosis in islet β-cells by reducing lipid uptake. Figure S7. rMSA upregulated glycolysis-related gene expression and increased glycolysis in hepatocytes. Figure S8. rMSA promoted glycolysis by upregulating the PI3K-AKT pathway in hepatocytes. Figure S9. rMSA upregulated the PI3K-AKT pathway through EGFR independently of IR or IGF-1R. [file 12967_2023_3957_MOESM1_ESM.pdf]

Additional file 1: Fig. S1

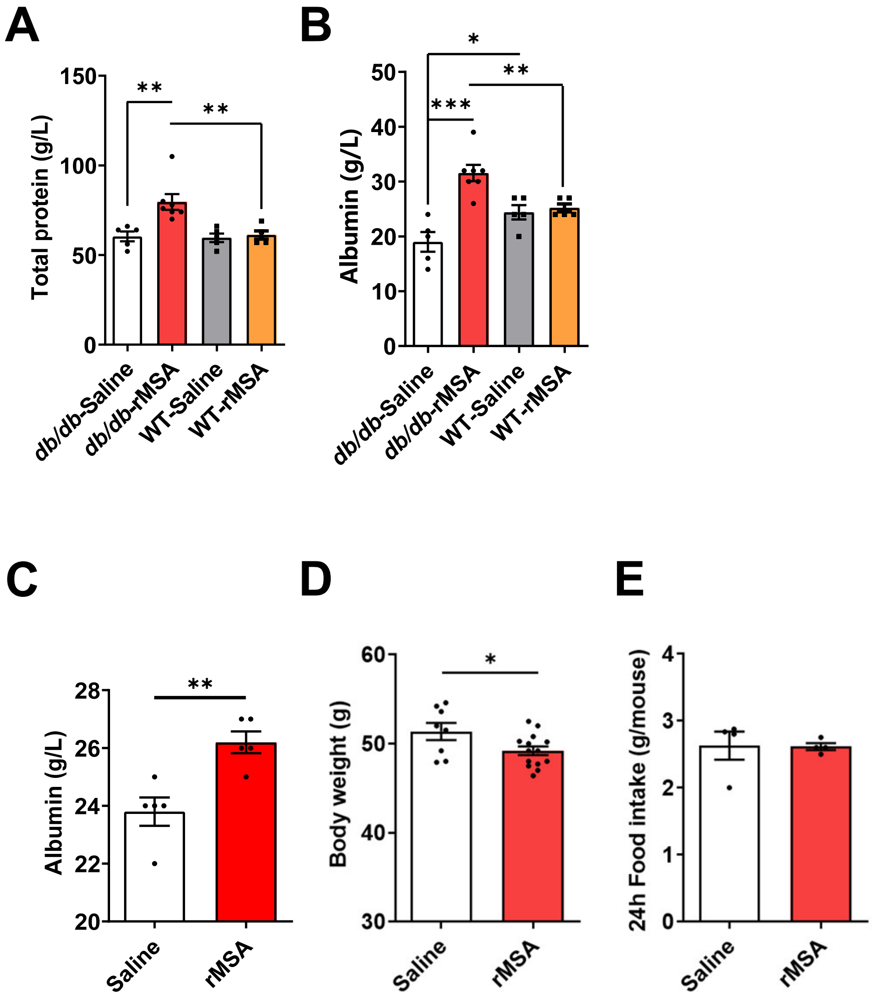

Additional file 1: Fig. S1. Therapeutic administration of rMSA increased total protein and serum albumin levels in T2DM mice.

(A) Serum total protein levels of *db/db* mice (n = 5-7 per group) and WT mice (n = 5 per group). The WT mice and *db/db* mice were treated with saline or rMSA for 9 weeks. (B) Serum albumin levels of *db/db* mice (n = 5-7 per group) and WT mice (n = 5 per group). (C) Serum albumin levels of DIO mice (n = 5 per group). (D) Body weight levels of DIO mice (n = 8-14 per group). (E) Food intake levels of DIO mice (n = 4 per group) in 24h. The WT mice, *db/db* mice and DIO mice were treated with saline or rMSA for 9 weeks. Data were analyzed by unpaired t-tests (A-E). Data are expressed as mean ± s.e.m. * $p < 0.05$, ** $p < 0.01$, *** $p < 0.001$.

Additional file 1: Fig. S2

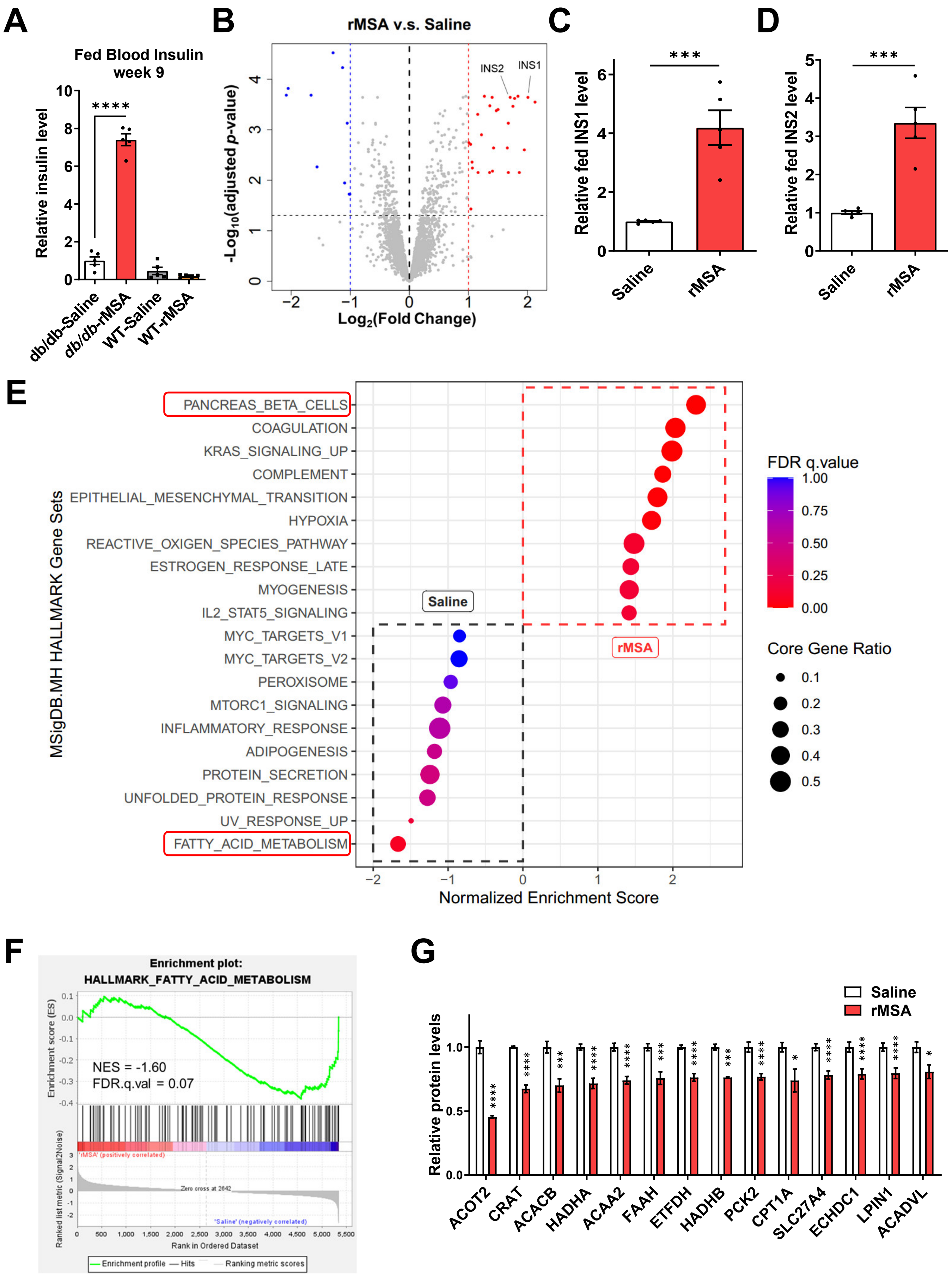

Additional file 1: Fig. S2. Therapeutic administration of rMSA increased the insulin level and decreased the fatty acid metabolism in the pancreas of T2DM mice.

(A) Relative fed blood insulin levels of *db/db* mice (n = 5-7 per group) and WT mice (n = 5 per group). The WT mice and *db/db* mice were treated with saline or rMSA for 9 weeks. (B) Volcanic map showing the protein levels identified by quantitative mass spectrometry in the pancreas of the *db/db* mice (n=5 per group) treated with the saline or rMSA for 9 weeks. The blue and red dots on the outside of the blue and red vertical dashed lines respectively show proteins with down-regulation and up-regulation by 2 times with an adjusted $p < 0.05$ (above the gray horizontal dashed line) in the rMSA group compared with the saline control. The mice were in a fed state before dissection. Relative levels of fed insulin 1 (C) and insulin 2 (D) were highlighted. (E) Bubble plot revealing enriched gene sets from GSEA results in protein levels identified by quantitative mass spectrometry in the pancreas of the saline- or rMSA-treated *db*/*db* mice (n=5 per group), according to MSigDB mouse-ortholog hallmark gene sets. The normalized enrichment score (NES), the ratio of genes contributing to the enrichment score, and the false discovery rate (FDR q.value) are shown. (F) Enrichment plot showing that the gene set of fatty acid metabolism is significantly enriched in the saline group, corresponding to (E). (G) Relative levels of proteins associated with fatty acid catabolism determined by quantitative mass spectrometry in the pancreas of the saline- or rMSA-treated *db*/*db* mice. Data were analyzed by unpaired t-tests (A, C, D, and G). Data are expressed as mean ± s.e.m. * $p < 0.05$, *** $p < 0.001$, **** $p < 0.0001$.

Additional file 1: Fig. S3

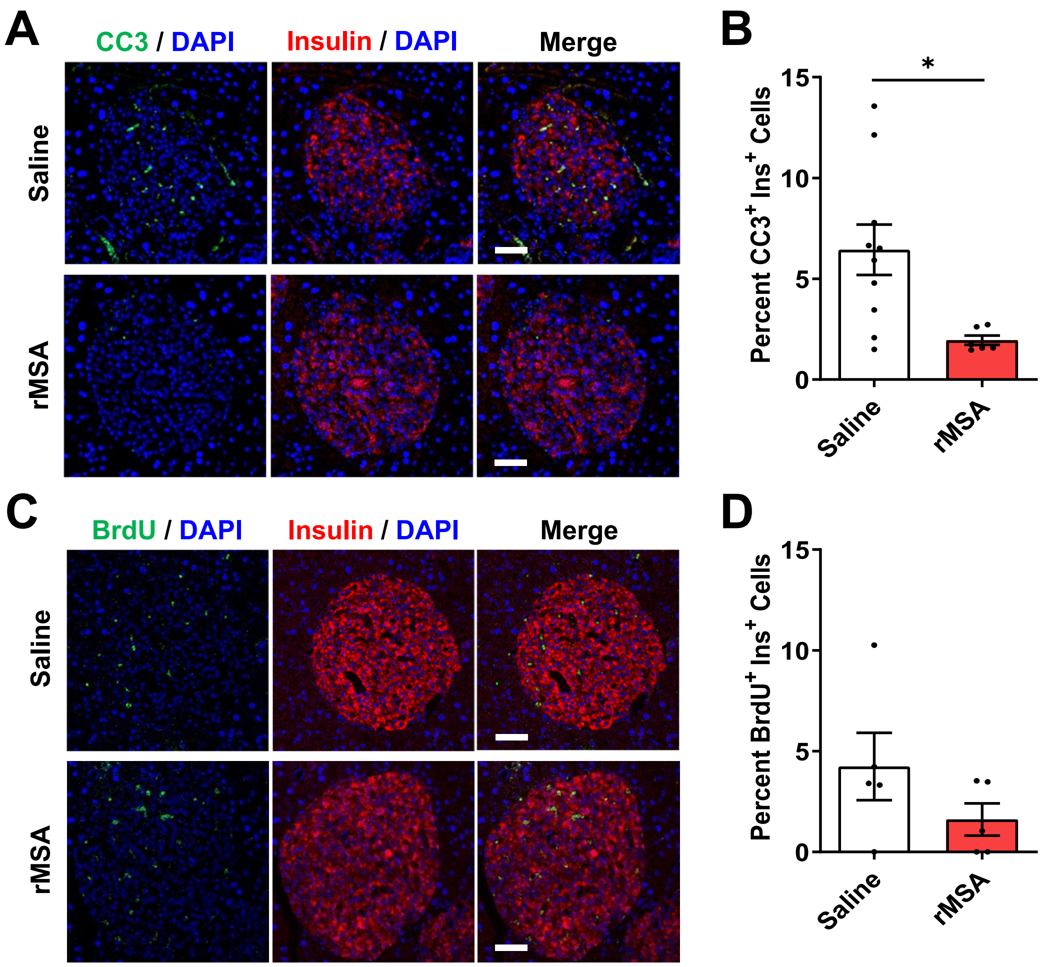

Additional file 1: Fig. S3. Therapeutic administration of rMSA prevented β-cell apoptosis in T2DM mice.

(A and C) Representative images of IF staining respectively for insulin, CC3, and BrdU in pancreases of the *db/db* mice treated with saline (CC3, n = 10; BrdU, n = 5) or rMSA (CC3, n = 6; BrdU, n = 5) for 9 weeks. Insulin staining is shown in red; DAPI-stained nuclei are shown in blue; CC3 (A) and BrdU (C) are shown in green. Scale bars represent 50 μm. (B and D) The percentage of indicated markers and insulin double-positive cells to all insulin-positive cells in the islets of the *db/db* mice treated with the saline or rMSA for 9 weeks, respectively corresponding to the representative images (A and C). Data were analyzed by unpaired t-tests (B and D). Data are expressed as mean ± s.e.m. * $p < 0.05$.

Additional file 1: Fig. S4

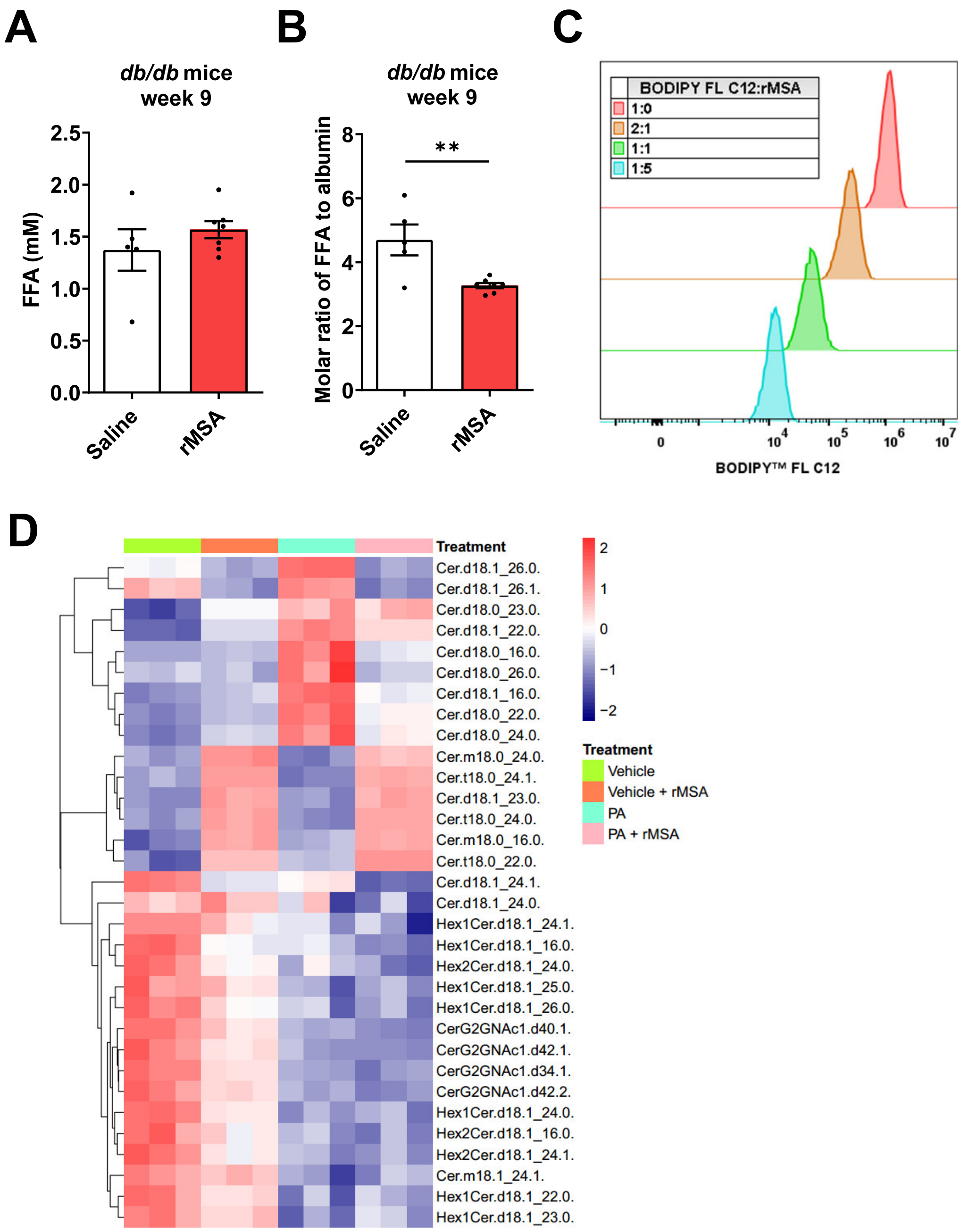

Additional file 1: Fig. S4. rMSA decreased the molar ratio of serum FFA to albumin in T2DM mice and reduced the FFA uptake in islet β-cells.

(A) Serum FFA levels of the *db/db* mice after the saline or rMSA treatments for 9 weeks. (B) The molar ratio of serum FFA to albumin in *db/db* mice treated with saline or rMSA for 9 weeks. (C) Representative histogram of the flow cytometry showing the fluorescent intensity under the indicated molar ratios of BODIPY™ FL C12 to rMSA. (D) Heat map showing the PA (0 or 0.2 mM) and rMSA (0 or 300 μM) treatments for 16 h on the intracellular relative ceramide levels of MIN6 cells. Data were analyzed by unpaired t-tests (A and B). Data are expressed as mean ± s.e.m. ** $p < 0.01$.

Additional file 1: Fig. S5

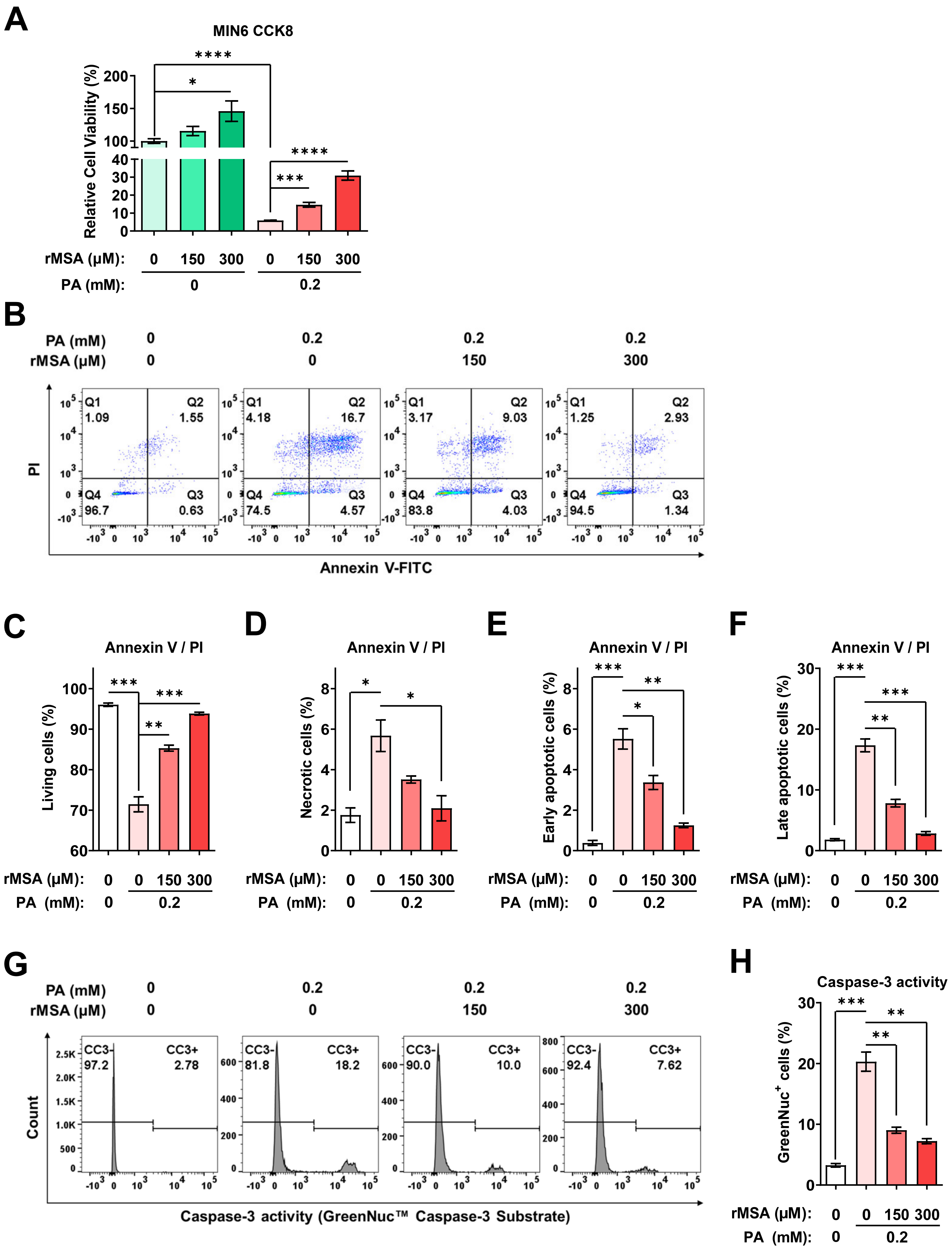

Additional file 1: Fig. S5. rMSA reduced lipotoxicity-induced islet β-cell apoptosis.

(A) Effects of PA and rMSA at indicated concentrations on MIN6 cell relative viability assessed by CCK-8 (n = 3-4 per group). (B) Representative flow cytometry analysis for Annexin V-FITC/PI in MIN6 cells treated with PA and rMSA at indicated concentrations (n = 3 per group). (C-F) Percentages of the living (C), necrotic (D), early apoptotic (E), and late apoptotic (F) MIN6 cells corresponding to (B). (G) Representative flow cytometry analysis for living-cell caspase-3 activity assays in MIN6 cells treated with PA and rMSA at indicated concentrations (n = 3 per group). (H) Percentages of the caspase-3-activated MIN6 cells, corresponding to (G). Data were analyzed by unpaired t-tests (A, C-F, and H). Data are expressed as mean ± s.e.m. * $p < 0.05$, ** $p < 0.01$, *** $p < 0.001$, **** $p < 0.0001$.

Additional file 1: Fig. S6

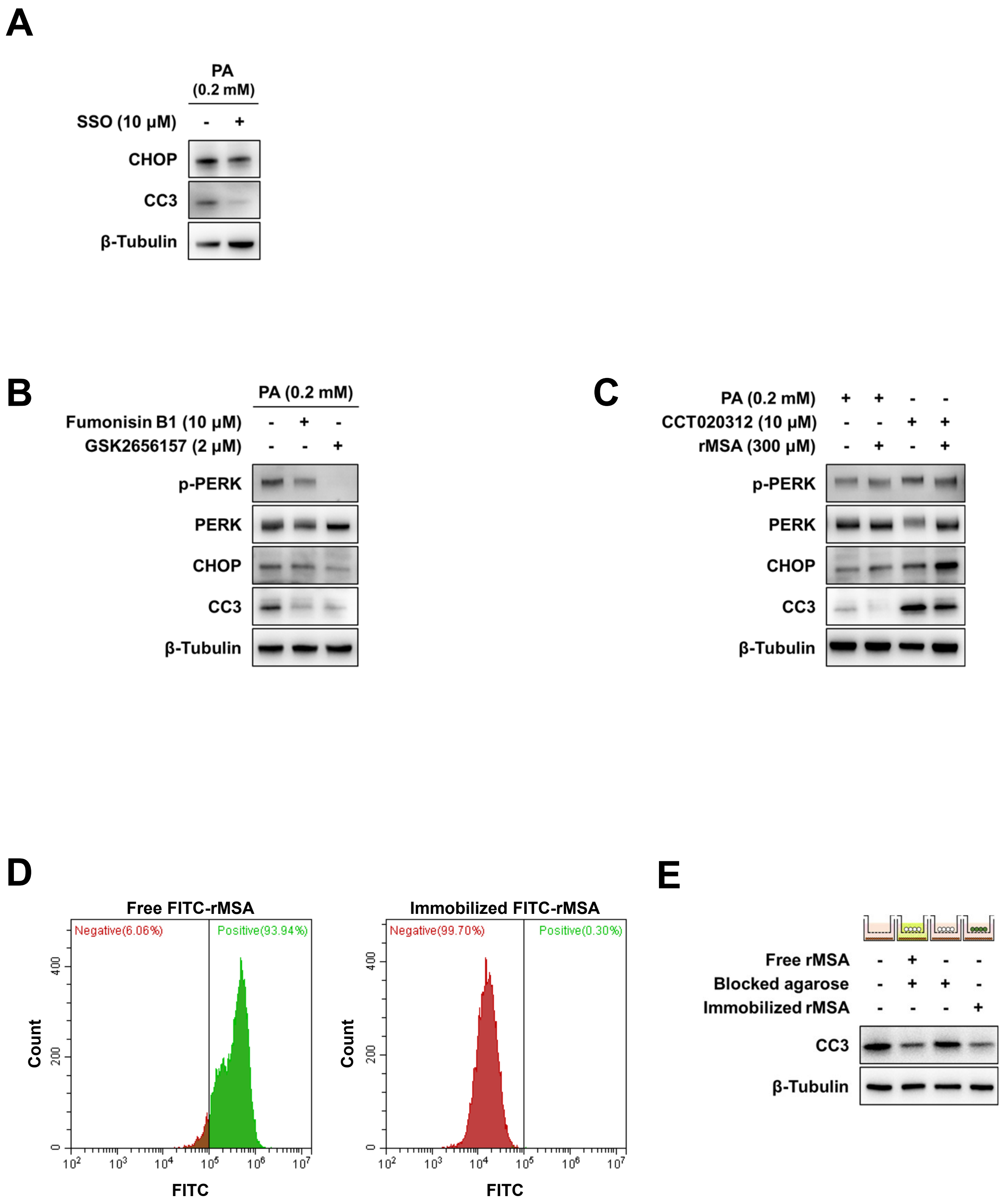

Additional file 1: Fig. S6. rMSA alleviates ER stress and apoptosis in islet β-cells by reducing lipid uptake.

(A) Western blot showing the protein levels of CHOP and CC3 in the MIN6 cells treated with vehicle or the CD36 inhibitor SSO (10 μM) in the presence of PA (0.2 mM). (B) Western blot showing the protein levels of phosphorylated PERK, PERK, CHOP, and CC3 in the MIN6 cells treated with vehicle, the ceramide synthetase inhibitor Fumonisin B1 (10 μM), or the PERK inhibitor GSK2656157 (2 μM) in the presence of PA (0.2 mM). (C) Western blot showing the protein levels of phosphorylated PERK, PERK, CHOP, and CC3 in the MIN6 cells treated with PA (0.2 mM), rMSA (300 μM), or the PERK agonist CCT020312 (10 μM). (D) Flow cytometry analysis for FITC intensity in MIN6 cells treated with free and immobilized rMSA. (E) Western blot showing the protein levels of CC3 in the MIN6 cells treated with PA (0.2 mM) and blank, free rMSA, blocked agarose, or immobilized rMSA. β-Tubulin was used as the internal reference (A-C and E).

Additional file 1: Fig. S7

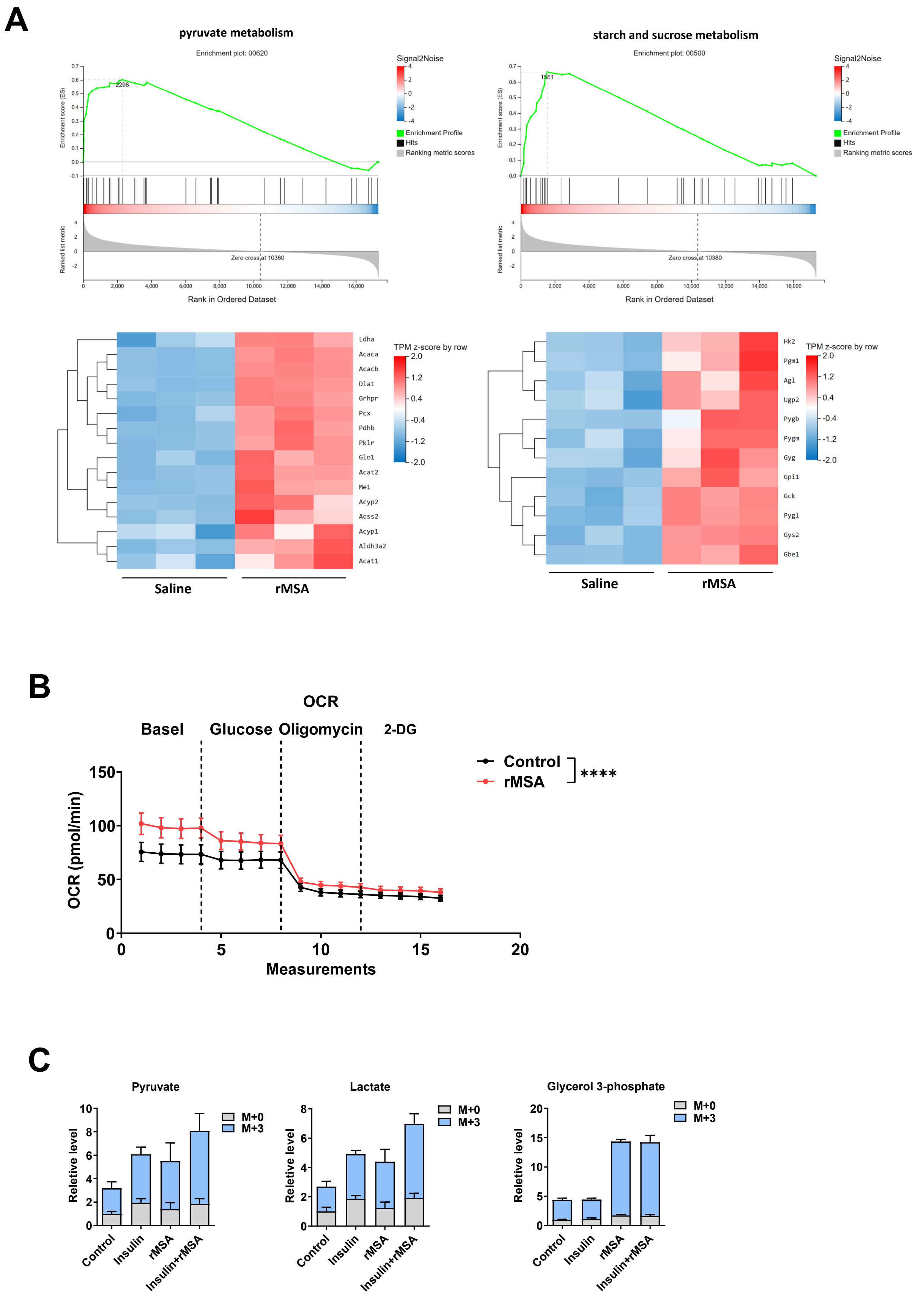

Additional file 1: Fig. S7. rMSA upregulated glycolysis-related gene expression and increased glycolysis in hepatocytes.

(A) Gene set enrichment analysis of up-regulated genes in livers of rMSA-treated *db/db* mice compared with the saline group (n = 3 per group). (B) Metabolic profile on AML12 hepatocytes (n = 11 per group) with or without rMSA (600 µM) treatment for 6h as measured by Seahorse glycolytic stress assay. (C) Isotope tracing of [U-13C]-glucose metabolism in AML12 hepatocytes (n = 3 per group) treated with vehicle, insulin (10 nM, 6h), rMSA (600 µM, 6h), and insulin (10 nM, 6h) +rMSA (600 µM, 6h). Data were analyzed by two-way ANOVA with repeated measures (B). Data are expressed as mean ± s.e.m. **** $p < 0.0001$.

Additional file 1: Fig. S8

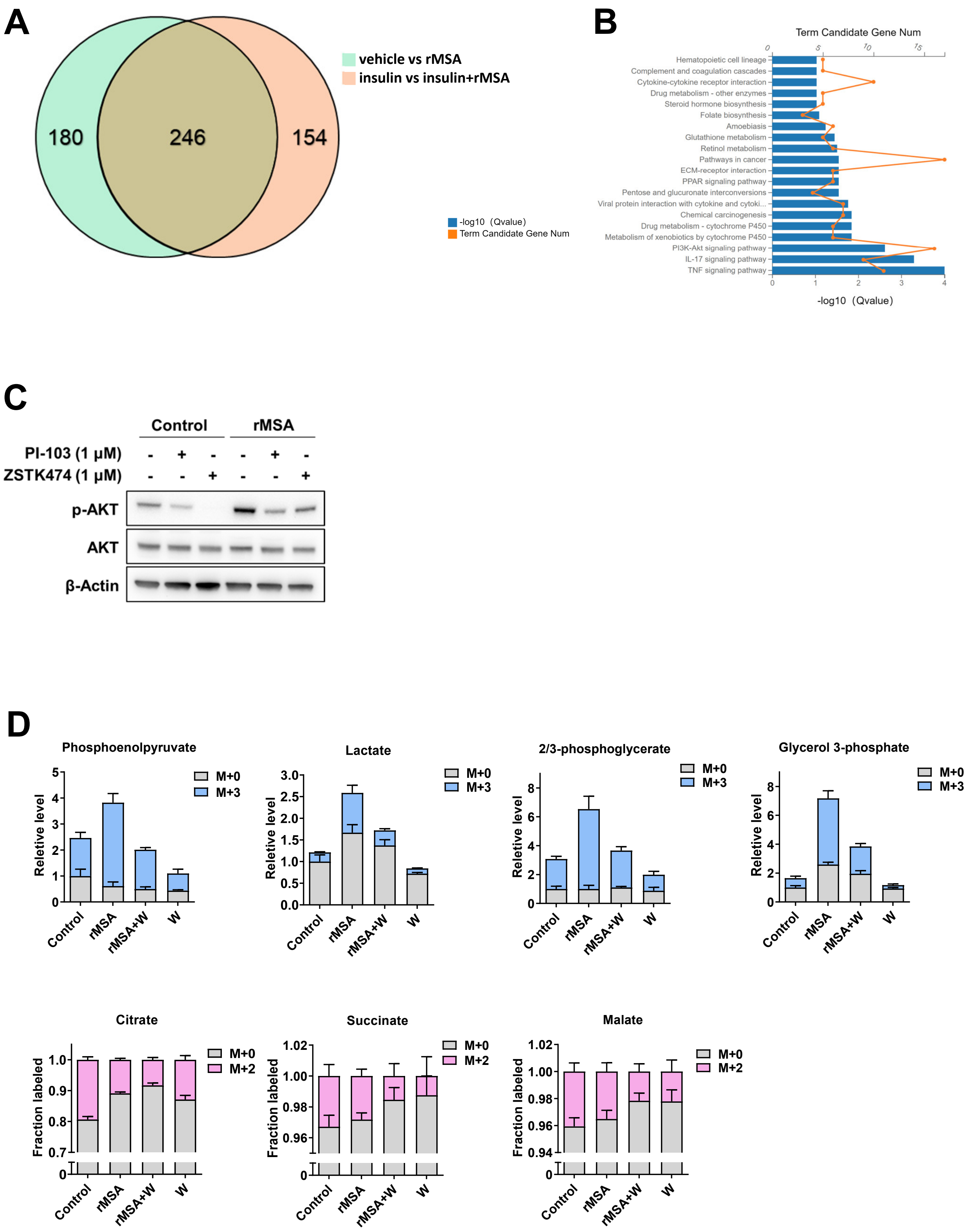

Additional file 1: Fig. S8. rMSA promoted glycolysis by upregulating the PI3K-AKT pathway in hepatocytes.

(A) Venn diagram showing the differentially expressed genes (DEGs) in AML12 hepatocytes (24 h, n = 3 per group) of different comparisons. $Log_2FC > 1$ and $p < 0.05$ were used to define the DEGs. (B) Pathway enrichment analysis of 246 DEGs from the comparisons of both "vehicle *v.s.* rMSA" and "insulin *v.s.* rMSA+insulin" (A). (C) Western blot showing the protein and phosphorylation levels of AKT in the AML12 hepatocytes treated with vehicle or rMSA (600 μM) in the absence or presence of 1μM PI-103 or 1μM ZSTK474 for 6h. β-Actin was used as the internal reference. (D) Isotope tracing of [U-13C]-glucose metabolism in AML12 hepatocytes (n = 3 per group) treated with vehicle or rMSA (600 μM, 6h) in the absence or presence of 1μM Wortmannin (W).

Additional file 1: Fig. S9

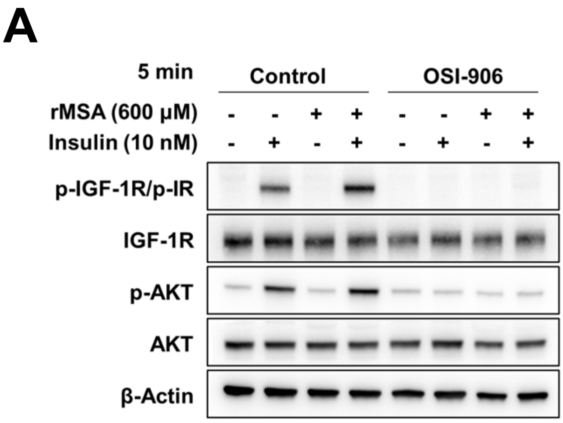

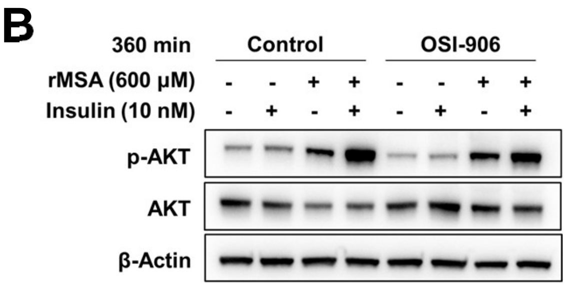

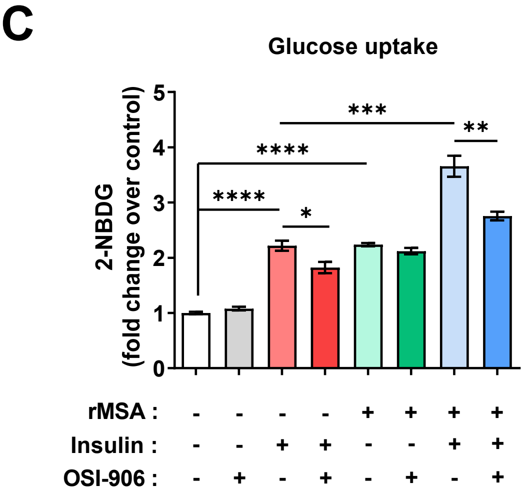

D

Term	Count	PValue	Fold Enrichment
mmu04012:ErbB signaling pathway	24	4.09217E-22	16.45
mmu04510:Focal adhesion	31	1.418E-20	8.93
mmu04010:MAPK signaling pathway	33	3.32686E-20	7.84
mmu04931:Insulin resistance	24	1.34757E-19	13.01
mmu04722:Neurotrophin signaling pathway	24	1.60049E-18	11.73
mmu04151:PI3K-Akt signaling pathway	35	1.18621E-17	5.95
mmu04150:mTOR signaling pathway	16	1.94211E-14	16.17

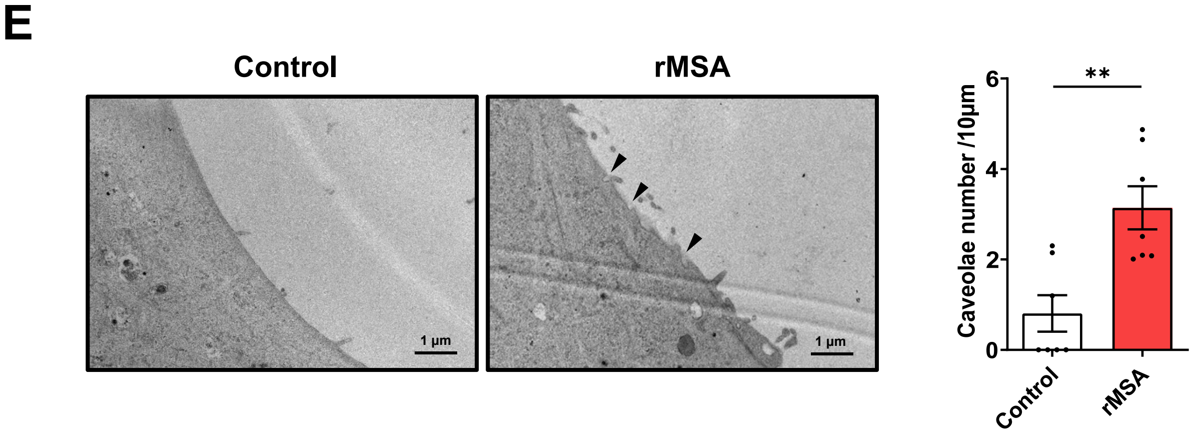

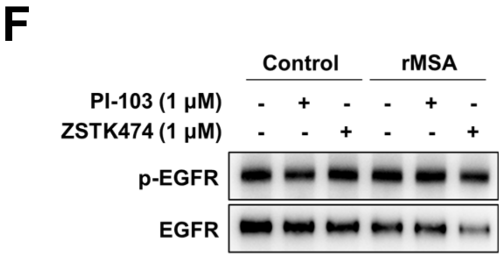

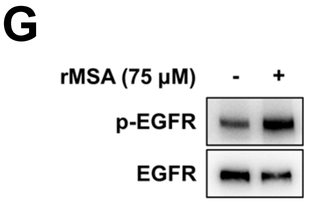

Additional file 1: Fig. S9. rMSA upregulated the PI3K-AKT pathway through EGFR independently of IR or IGF-1R.

(A) Western blot showing the protein and phosphorylation levels of IGF-1R and AKT in the AML12 cells treated with vehicle or rMSA (600 μM), with or without insulin (10 nM) for 5 min in the absence or presence of 1μM OSI-906. (B) Western blot showing the protein and phosphorylation levels of AKT in the AML12 cells treated with vehicle or rMSA (600 μM), with or without insulin (10 nM) for 360 min in the absence or presence of 1μM OSI-906. (C) 2-NBDG uptake in AML12 hepatocytes (n = 3-4) treated with vehicle or rMSA (600 μM, 6h), with or without insulin (10 nM, 1h) in the absence or presence of 1μM OSI-906. (D) Pathway enrichment analysis of upregulated protein phosphorylation levels in rMSA-treated AML12 hepatocytes compared with the vehicle-treated cells. (E) The AML12 hepatocytes with or without rMSA treatment (600 μM, 2h) were fixed and visualized by transmission electron microscopy. Arrowheads indicate caveolae. (Scale bar. 1 μm.). The number of caveolae structures on the cell membrane was quantified. (F) Western blot showing the protein and phosphorylation levels of EGFR in the AML12 hepatocytes treated with vehicle or rMSA (600 μM) for 6h in the absence or presence of 1μM PI-103 or 1μM ZSTK474. (G) Western blot showing the protein and phosphorylation levels of EGFR in the mouse primary hepatocytes treated with vehicle or rMSA (75 μM) for 24h. Data were analyzed by unpaired t-tests (C and E). Data are expressed as mean ± s.e.m. * $p < 0.05$, ** $p < 0.01$, *** $p < 0.001$, **** $p < 0.0001$. β-Actin was used as the internal reference (A and B).
